# The underlying mechanical properties of membranes tune their ability to fuse

**DOI:** 10.1016/j.jbc.2023.105430

**Published:** 2023-11-04

**Authors:** Rafael B. Lira, Jayna C.F. Hammond, Rafaela R.M. Cavalcanti, Madelief Rous, Karin A. Riske, Wouter H. Roos

**Affiliations:** 1 Moleculaire Biofysica, Zernike Instituut, Rijksuniversiteit Groningen, Groningen, Netherlands; 2 Departamento de Biofísica, Universidade Federal de São Paulo, São Paulo, Brazil

**Keywords:** membrane fusion, viscosity, bending rigidity, edge tension, membrane mechanics, cholesterol, membrane pores

## Abstract

Membrane fusion is a ubiquitous process associated with a multitude of biological events. Although it has long been appreciated that membrane mechanics plays an important role in membrane fusion, the molecular interplay between mechanics and fusion has remained elusive. For example, although different lipids modulate membrane mechanics differently, depending on their composition, molar ratio, and complex interactions, differing lipid compositions may lead to similar mechanical properties. This raises the question of whether (i) the specific lipid composition or (ii) the average mesoscale mechanics of membranes acts as the determining factor for cellular function. Furthermore, little is known about the potential consequences of fusion on membrane disruption. Here, we use a combination of confocal microscopy, time-resolved imaging, and electroporation to shed light onto the underlying mechanical properties of membranes that regulate membrane fusion. Fusion efficiency follows a nearly universal behavior that depends on membrane fluidity parameters, such as membrane viscosity and bending rigidity, rather than on specific lipid composition. This helps explaining why the charged and fluid membranes of the inner leaflet of the plasma membrane are more fusogenic than their outer counterparts. Importantly, we show that physiological levels of cholesterol, a key component of biological membranes, has a mild effect on fusion but significantly enhances membrane mechanical stability against pore formation, suggesting that its high cellular levels buffer the membrane against disruption. The ability of membranes to efficiently fuse while preserving their integrity may have given evolutionary advantages to cells by enabling their function while preserving membrane stability.

Membrane mechanics plays a crucial role in a variety of biological processes, such as cell motility, exocytosis, and division (1, 2). In the cell, membranes are constantly undergoing remodeling, budding, and fusion (3, 4). The mechanical properties of membranes are determined by factors that include the lipid composition, membrane proteins, and cytoskeletal interactions. While the last two have been widely studied (5, 6), the role of lipid composition is by far less understood. More specifically, how the lipid composition, and by extension its effects on membrane mechanics, determines the ability of membranes to fuse is not completely known. Not surprisingly, the membrane composition of the various intracellular organelles varies considerably and so do their mechanical properties (7, 8, 9). For instance, while the endoplasmic reticulum is rich in shorter, unsaturated and apolar lipids, exocytic vesicles and the plasma membrane (PM) are rich in long, saturated and charged lipids, with sorting organelles such as Golgi having an intermediate composition (8, 9). This variety in composition in the secretory pathway creates intracellular territories with a gradient in membrane curvature, packing defects, and electrostatics that have wide biological implications (8). The best studied biomembrane is the PM. The PM is compositionally asymmetric, with sphingolipids enriched in the outer leaflets, whereas the inverted cone-shaped lipid phosphatidylethanolamine and the charged phosphatidylserine (PS) are enriched in the inner leaflet (7, 10). Furthermore, lipids in the inner leaflet contain about twice as many lipids with unsaturation in their acyl chains (10). These differences in compositions give rise to large biophysical differences across both leaflets, with the outer leaflet being more packed (*i.e.* ordered) and the inner more fluid (10, 11).

Membrane fusion, a process of merging two initially separated membranes, is one of the many biological processes that is largely dependent on membrane mechanics (12, 13). Despite major differences in kinetics and locations in the cell, fusion follows several well-defined and conserved intermediates. It starts by protein-mediated membrane interactions and local deformation (14, 15, 16), after which a highly curved fusion diaphragm, or stalk, is formed. Due to its high curvature, the stalk is favored in more fluid membranes as well as in membranes containing negatively-curved lipids (17, 18). The rupture of the fusion diaphragm and the formation of a fusion pore results in the mixing of the inner leaflets (13, 14, 18, 19). The expansion of the fusion pore leads to the collapse of the fusing membranes and mixing of the initially separated aqueous compartments, that is, when full-fusion occurs (13, 19, 20, 21). It has been experimentally observed, both *in vivo* and *in vitro*, that mixing of inner leaflet lipids are not necessarily accompanied by mixing of the aqueous compartments (22, 23). This is mainly because in certain conditions, the fusion pore may be too small for the passage of large aqueous probes through the (narrow) fusion pores (21). Thus, when studying fusion, it is important to resolve whether fusion occurs only through lipid mixing or also *via* content mixing (24).

At the PM, fusion can start at either of its leaflets; intracellular vesicles contact and fuse with the PM from the inner leaflet (25), while some viruses readily fuse with the PM from the outer leaflet side (26). Recent molecular dynamics simulations revealed that, due to the high fraction of unsaturated lipids and high fluidity, the inner leaflet of the PM is far more fusogenic than the outer leaflet (27). This is a result of the high energies associated with outer leaflet stalk formation. In any case, since these membranes undergo full fusion, both leaflets must be permissive to fusion despite large differences in the underlying mechanics. Because fusion involves transits through highly curved intermediates, it is expected that stiffer membranes, which offer higher resistance to deformation, would be less amenable to fusion, explaining the high fusogenic nature of the inner leaflet of the PM. In fact, it has been widely hypothesized that an increase in membrane rigidity reduces fusion efficiency, whereas softening membranes improves fusion (13, 19). A striking example comes from virus fusion. HIV virus infection is reduced upon treatment with the antiviral drug Serinc5, which acts by inhibiting pore formation and dilation. Exogeneous incorporation of phosphatidylethanolamine lipids rescues fusion by softening the viral envelope (28). Of note, HIV virus-like particles fuse with membranes rich in cholesterol (Chol) (29), and fusion is largely favored by Chol-dependent lateral domains and line tension (30). This suggests a less specific effect of Chol but rather an effect that depends on the domain line tension (30, 31). In fact, fusion is facilitated in membranes that exhibit domains even in the absence of Chol (32), suggesting that membrane mechanics may, in some cases, play a more important role than the specific lipid composition.

Chol is an interesting molecule in the context of membrane fusion. While on the one hand, it tends to increase membrane viscosity (33), packing (34), and stiffness (35), presumably making membranes less prone to fusion, its inverted conical shape fits well within the fusion stalk, and thus Chol is expected to favor fusion (36). However, it has been recently shown that for Ebola virus, Chol helps fusion not by necessarily modulating membrane mechanics but instead by interacting with viral factors (37). Importantly, the addition of Chol makes membranes more mechanically resilient, renders membranes more impermeable to water (38), and results in higher lysis (39) and edge tension (40). Additionally, it has been hypothesized that the high packing of the outer leaflet imparts to the greater mechanical resistance and limited permeability of the PM (10). In fact, fusion-dependent disruption has been observed in several contexts (41, 42, 43). Thus, it seems that both the ability of membranes to fuse as well as their mechanical resilience against disruption may be correlated. In fact, membranes made of grafted copolymers, which make the membranes significantly softer than their lipid counterparts (44), are more fusogenic (44, 45). However, these very fluid membranes are also more prone to disruption as a result of their low edge tension (45). Hence, although Chol seems to be an important modulator of fusion and membrane fusion as well as disruption, there are clear indications that these processes depend on more general mechanical effects rather than on the specific lipid composition.

To address these somewhat conflicting results, we performed a systematic study to characterize the role of the underlying mechanics on membrane fusion and fusion-mediated membrane disruption. As a fusion system, we use a recently developed assay based on giant unilamellar vesicles (GUVs) and fusogenic large unilamellar vesicles (LUVs) (46). This system is studied by a combination of single vesicle time-resolved fluorescence resonance energy transfer (FLIM-FRET), confocal multicolor content mixing, and membrane permeabilization assays. Confirming previous theoretical findings, we show that fusion is favored with membranes mimicking the inner leaflet of the PM due to a combination of high fluidity and electrostatic interactions. To separate these two factors, we tune the mechanics of membranes of identical charge by preparing very fluid membranes made of low melting temperature (Tm) lipids, with or without Chol, and solid membranes made of high Tm lipids, as well as the intermediate liquid-ordered (Lo) membrane made of mixtures of high Tm lipids with Chol. We use (i) fluorescence lifetime imaging microscopy (FLIM) combined with molecular rotors to measure membrane viscosity, (ii) electroporation and fast imaging recording to measure edge tension from pore closure dynamics, and we (iii) correlate these properties with published data from membrane bending moduli for the relevant compositions. We show that membrane viscosity and stiffness are highly correlated and dependent on nontrivial and combined effects of charge and Chol; however, these properties are not correlated with edge tension. Importantly, fusion becomes progressively more efficient as membrane fluidity increases irrespective of the specific lipid composition. Chol mildly reduces fusion efficiency but strongly increases the resilience of membranes against fusion-dependent membrane disruption. The findings shed new light onto the more universal effects of membrane mechanics on modulating membrane fusion and the downstream effects of fusion on membrane stability.

## Results

**Figure 1:**
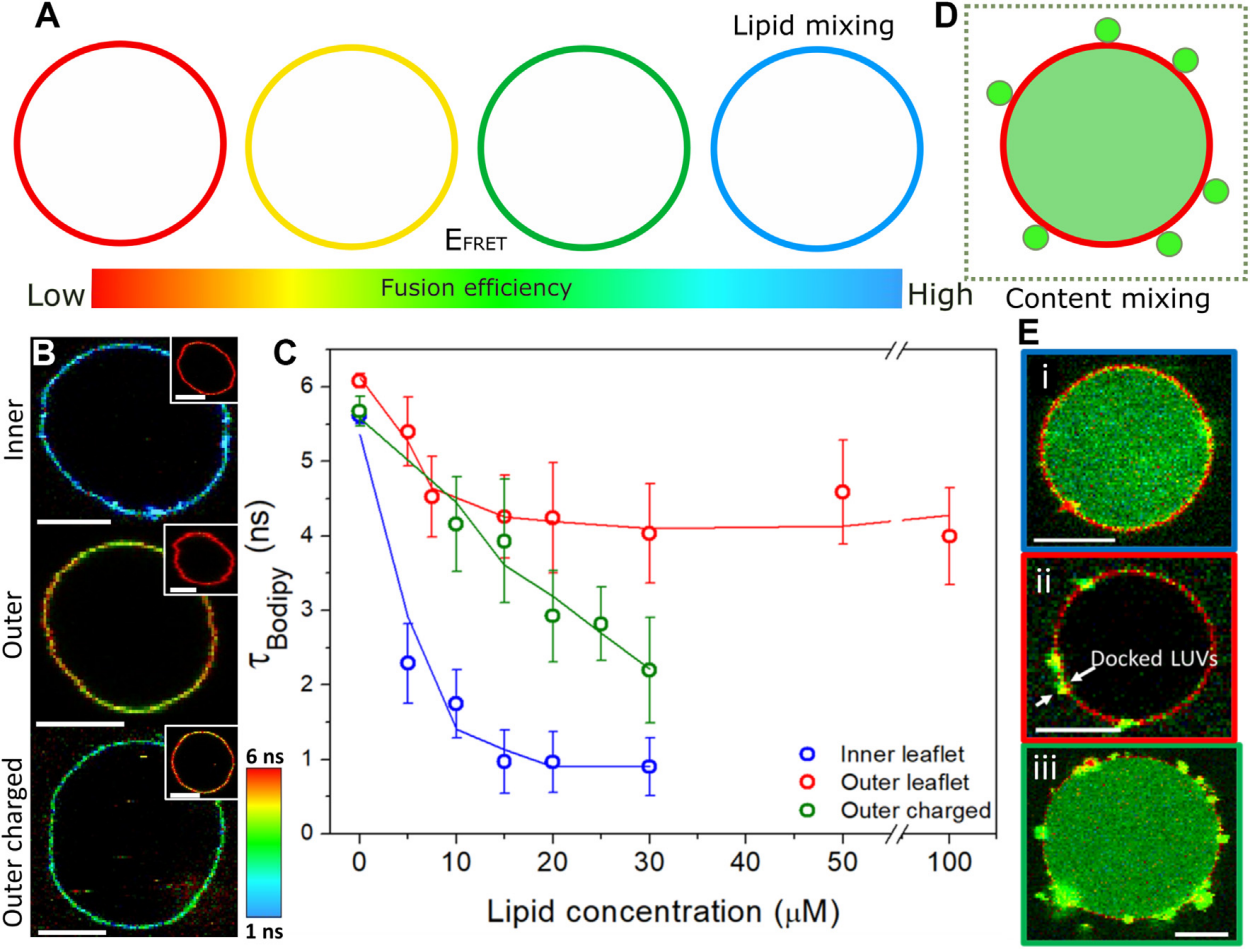
**A FLIM-FRET fusion assay shows that fusion of cationic LUVs is more efficient with PM inner leaflet membrane mimic.***A*, a sketch of FLIM-FRET lipid-mixing assay. As more FRET-acceptor LUVs fuse with FRET-donor GUVs, the donor lifetime measured in the GUVs shortens, as represented by a lifetime color scale, yielding higher FRET efficiencies (E_FRET_). *B*, representative images of inner, outer, and outer charged leaflet mimic GUVs incubated with 20 μM LUVs. Insets show respective control GUVs without LUVs. Bars represent 10 μm. *C*, LUV concentration– (total lipid) dependent changes in GUV donor lifetime for the three membrane mimics. All controls (0 μM LUVs) were fitted with one exponential decay, whereas samples incubated with the LUVs were fitted with two decays, from which the weighted average was used, with 10 to 15 GUVs/composition analyzed. *D*, a sketch of the content-mixing assay, with LUVs encapsulating the water-soluble SRB probe docking and fusing with a single GUV (*red*). Docking is detected by the diffraction-limited LUV spots on the GUV surface, whereas content mixing is detected by the appearance of SRB signal in the GUV interior. *E*, representative images of inner (i), outer (ii), and outer charged (iii) GUV mimics in the content-mixing assay, respectively. *Arrows* indicate docked LUVs. The results represent the general trend observed for 10 to 15 GUVs/composition. The *red* GUV membrane in Figure E is a result of the dim Bodipy C_16_ signal detected upon SRB excitation. Scale bars represent 7 μm. FLIM, fluorescence lifetime imaging microscopy; GUV, giant unilamellar vesicle; LUV, large unilamellar vesicle; PM, plasma membrane; SRB, sulforhodamine B.

We started by adapting the LUV-GUV fusion assay (46) to study lipid mixing and extended it to probe content mixing and membrane permeabilization using FLIM. In short, when LUVs containing the FRET acceptor dye DPPE-Rh fuse with GUVs labeled with the donor probe Bodipy C_16_, the fluorescence lifetime of the donor probe is shortened due to FRET. Changes in lifetime are detected at the single pixel level using time-correlated single photon counting and used to assess E_FRET_, a quantitative measure of fusion efficiency (Fig. 1*A*). We use Bodipy C_16_ as donor due to its long and single exponential decay that is insensitive to environmental factors such as membrane polarity (Lira *et al.*, submitted), and thus changes in donor lifetime are exclusively associated with FRET. Separately, we performed a content mixing assay using multicolor confocal microscopy to assess the fusion of LUVs encapsulating the water-soluble probe Dextran-FITC 3 kDa and its transfer into the aqueous compartment of the GUV (Fig. 1*E*). A decrease in donor lifetime (high E_FRET_) accompanied by Dextran-FITC 3 kDa transfer is indicative of full-fusion, whereas changes in E_FRET_ without Dextran-FITC 3 kDa transfer is indicative of hemifusion. Since the LUVs can be visible as diffraction-limited spots, the assay also resolves membrane docking. To investigate fusion-dependent membrane disruption, we performed two leakage assays: we used FLIM to resolve two spectrally similar dyes but with clearly resolvable fluorescence lifetimes or we used multicolor confocal microscopy with two spectrally distinct dyes to simultaneously assess fusion and leakage. In the first assay, we assessed membrane permeabilization and the size of the formed pores by studying the entry of the small fluorescent probe KU530 (∼0.65 kDa) or of the large probe Dextran-FITC (3 kDa) upon fusion. Alternatively, DOPE-Atto647N (hereby Atto) present in the LUV membrane as a fusion marker and SRB present in the outside medium as the leakage marker. In this case, fusion is detected as the transfer of Atto from the LUVs to the GUV membranes, whereas SRB was used as a membrane impermeable molecule. KU530, Dextran 3 kDa, or SRB are present exclusively outside the GUVs if their membranes are intact. However, if the integrity of the GUV membrane is compromised (*i.e.* presence of membrane pores), there is an exchange of the internal and external aqueous solution and dye leakage to the GUV interior.

### Fusion with inner and outer leaflet mimics of the PM

We started by investigating the role of the PM leaflet composition on fusion using FLIM-FRET. As a membrane model, we used GUVs composed of DOPC:PS:Chol:DOPE, or alternatively DOPG instead of PS, (25:25:25:25) mol as an inner leaflet mimic, and GUVs composed of SM:DOPC:PS:DOPE:Chol (24:30:0.6:5.4:40, mol ratio) as the outer leaflet mimic (11). Although the GUVs produced are symmetrical, unlike the PM, the experiments are useful as they separate the effects of individual leaflet composition on fusion. Thanks to their composition, the inner leaflet membranes are charged and highly fluid, whereas outer leaflet membranes, rich in SM and Chol, are nearly neutral and highly viscous. We thus anticipate the inner leaflet mimic to be more permissive to fusion with the cationic LUVs than its outer leaflet counterpart. As expected, fusion is significantly more efficient with inner leaflet mimics (Fig. 1, *B* and *C*) and it occurs *via* content mixing (Fig. 1*E*, (i). For these membranes, fusion saturates at 15 to 20 μM LUV concentration. Concentrations at and above 50 μM results in GUV collapse and were not included in the analysis. With outer leaflet GUV mimics, LUV fusion produces much milder shortening of donor lifetime (*i.e.* fusion) that already saturates at ∼5 μM LUVs, presumably due to the quick consumption of low charges in these membranes (0.6 mol% anionic lipids), although the LUVs efficiently dock to the these GUVs (arrows in Fig. 1*E*, (ii). This suggests an effect on fusion, not docking. Not surprisingly, the very limited fusion is not followed by full fusion. The results are in close agreement with molecular dynamic simulation (27) and are interpreted as a result of the combination of low charge and high stiffness of outer leaflet membranes.

To disentangle the effects of membrane fluidity and charge, we prepared GUVs containing a composition similar to that of the PM outer leaflet but containing the same fraction of anionic lipids of that in the inner leaflet. Figure 1, *B* and *C* show that fusion occurs efficiently with these membranes but to a lower extent when compared to the inner leaflet mimic. In fact, the extent of fusion as assessed by donor quenching is roughly intermediate between the two leaflet mimics, suggesting that these factors have approximately equal contributions for fusion. Importantly, fusion with these membranes occur *via* full fusion (Fig. 1*E*, (iii). The results show that both charge and membrane fluidity are important factors determining membrane fusion efficiency in our system.

**Figure 2:**
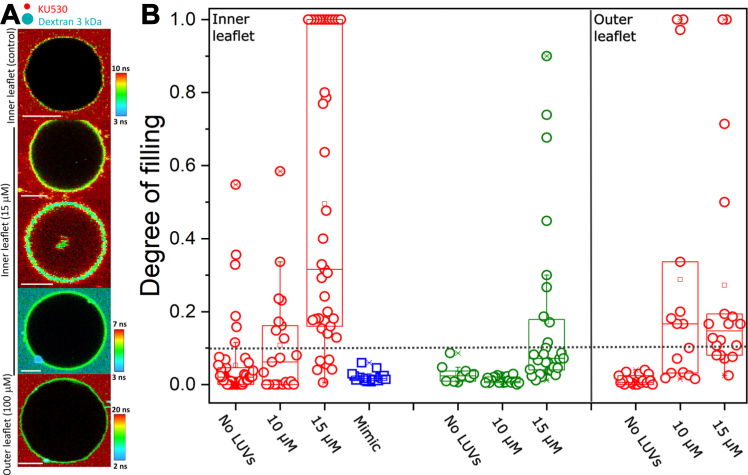
**Fusion-dependent membrane disruption of plasma membrane leaflet mimics.***A*, representative FLIM images of inner or outer leaflet membrane mimics in the presence of different concentrations of LUVs (lipid-based) co-incubated with KU530 (0.02 mg/ml) or dextran 3 kDa (0.01 mg/ml) as markers for small or large pore formation. The GUVs were labeled with 0.5 mol% Bodipy C_16_. Note that due the leakage and membrane dyes have similar spectral properties (*i.e.* color) but are distinguishable based on their differences in fluorescence lifetime. Scale bars represent 6 μm. *B*, degree of filling measured for individual GUVs as a function of (non-labeled) LUV concentration for the different GUV compositions tested co-incubated in the presence of KU530 (*red*) or dextran 3 kDa (*green*). We also included the inner mimic membranes whose composition (DOPC:PS:DOTAP:Chol:DOPE, 0.17:0.17:0.17:0.17:0.32) mimics that expected upon fusion of LUVs up to complete charge saturation. The *dashed line* indicates the arbitrarily-defined background in leakage. DOPC, 1,2-dioleoyl-sn-glycero-3-phosphocholine; DOPE, 1,2-dioleoyl-sn-glycero-3-phosphoethanolamine; DOTAP, 1,2-dioleoyl-3-trimethylammonium-propane; FLIM, fluorescence lifetime imaging microscopy; GUV, giant unilamellar vesicle; LUV, large unilamellar vesicle; PS, phosphatidylserine.

**Supporting Figure S1:**
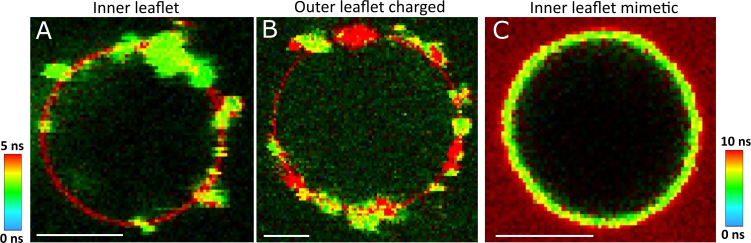
**Indication of permeabilization of plasma membrane mimics.** Figures *A* and *B* show representative images of inner and outer leaflet mimics GUVs during the content mixing experiment. The transferred content marker Dextran 3 kDa is lost likely due to the formation of membrane pores. *C*, a representative GUV whose composition mimics that of the inner leaflet mimics of the plasma membrane after fusion with LUVs up to charge saturation (DOPC:PS:DOTAP:Chol:DOPE, 0.17:0.17:0.17:0.17:0.32). The experiments were performed in the presence of 0.02 mg/ml KU530. Scale bars represents 9 μm.

To assess whether fusion with these membranes leads to membrane disruption, we incubated the inner or outer leaflet mimics GUVs with nonlabeled LUVs and in the presence of either the KU530 or Dextran-3 kDa as markers of small or large pores, respectively. We chose these two GUV compositions as they are the biologically relevant mimics of the inner or outer leaflets of the PM. In the lifetime map range selected, the GUV membrane (labeled with Bodipy C_16_) looks green-yellow, whereas KU530 and Dextran 3 kDa look red or turquoise, respectively. As shown in Figure 2*A*, control GUVs before incubation with the GUVs are intact (no dye signal inside). In contrast, incubation with 15 μM LUVs (concentration of lipids) leads to permeabilization of a fraction of the inner, but not the outer membrane mimics, and with this LUV concentration, only the small probe permeates through the membrane. To quantitatively assess fusion-dependent disruption, we performed the degree of filling analysis (47), in which permeabilization can be classified as (i) graded, wherein probe entry does not reach full equilibration, or as (ii) all-or-none, wherein entry has achieved full equilibration. In other words, the degree of filling (DOF) informs on the extent of fusion-dependent membrane disruption, with higher DOF being a result of more extensive disruption (*i.e.* larger and/or more pores open for longer periods). We arbitrarily defined DOF 0.1 as the threshold for permeabilization. As observed in Figure 2*B*, disruption is LUV-concentration dependent, with a larger fraction of GUVs becoming permeable which is also accompanied by a higher degree of pore entry at increasing LUV concentration. This indicates either the formation of larger or longer-lasting pores. In fact, when using Dextran 3 kDa as a disruption marker, one can see that the GUVs only become significantly permeable at higher LUV concentrations, indicating the enlargements of the formed pores. One could argue these may be a consequence of the final GUV composition (upon merging of LUV lipids with the GUVs) rather than as a consequence of fusion. This is ruled out by showing that GUVs with the expected mimic composition after fusion are intact, thus demonstrating that it is the fusion process that leads to pore formation. Not surprisingly, the outer mimics of the PM that are not permissive to fusion also are virtually impermeable even at much higher LUV concentrations. Although we did not perform a complete characterization with the outer charged mimics given its lack of biological relevance, we noted that some GUVs lost the accumulated content marker upon full-fusion (Fig. S1), suggesting that these membranes may also become permeabilized and indicates that fusion and permeabilization are coupled. In summary, the fluid and charged membranes of the inner leaflet are more permissive to fusion than their outer counterpart, extensive fusion leads to permeabilization and enlargement of the formed pores at higher extents of fusion, whereas the nonpermissive outer leaflet mimics do not become permeable. Importantly, it is the fusion process, not the changes in membrane composition that permeabilize membranes, pointing out to the coupling between (extensive) fusion and disruption.

### Mechanical characterization of the fusing membranes

**Figure 3:**
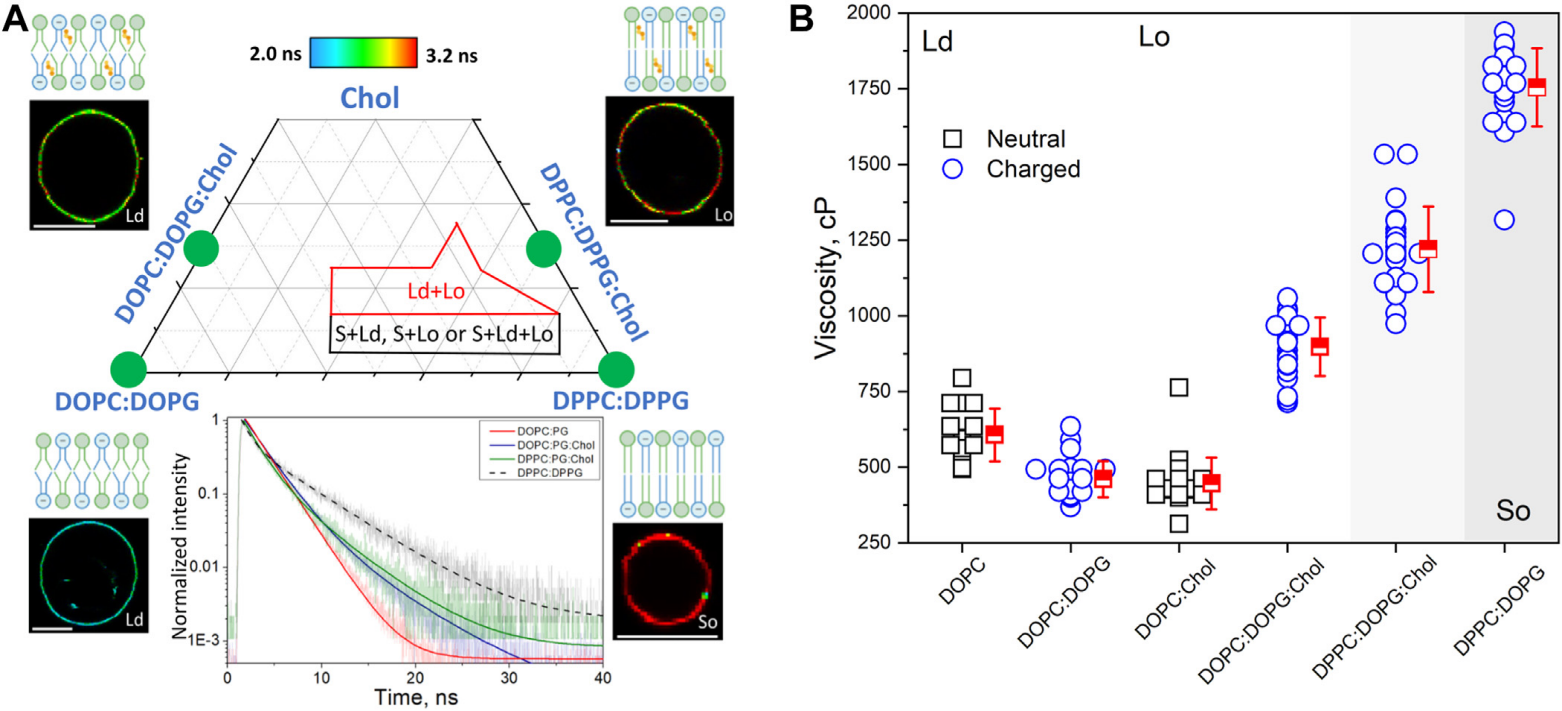
**Membrane viscosity on neutral and charged membranes in different phase states.***A*, phase diagram of DOPG:eSM:Chol adapted from (47). Ld, Lo, and So represent membranes in the liquid disordered, liquid ordered, and solid phase, respectively. The regions represent compositions in the diagram where the membrane exhibit two or more phases, whereas in the region outside the membrane is homogeneous. The solid *green* circles represent the compositions studied here. The insets show representative FLIM images of GUVs of each of these compositions. Scale bars represent 9 μm. The fluorescence decays are best fit with a two-exponential model and are also shown. The sketches represent the expected membrane organization. *B*, calculated viscosity from the long decay of Bodipy C_16_ was assessed from the calibration curve as in61 for neutral (*black squares*) and negative (*blue circles*) membranes. Each point represents a measurement on an individual GUV. Means and S.D. are also shown. The different membrane phases are shown as different *background shades*. All GUVs were labeled with 0.5 mol% of the molecular rotor Bodipy C_12_. DOPG, 1,2-dioleoyl-sn-glycero-3-phospho-(1′'-rac-glycerol); FLIM, fluorescence lifetime imaging microscopy; GUV, giant unilamellar vesicle; Ld, liquid disordered.

LUV fusion with mimics of the PM leaflets depends on both membrane fluidity and charge. To separate these two factors, we prepared GUVs of defined compositions and in different physical states but all containing the same amount of charged lipids, so that fluidity is the only relevant parameter in the experiments. For this, we prepared GUVs in different physical states and thus exhibiting very distinct mechanical properties; two very fluid liquid-disordered (Ld) GUVs made of (i) DOPC:DOPG (5:5 mol ratio), (ii) DOPC:DOPG:Chol (2:5:3 mol ratio), a Lo GUV composition made of (iii) DPPC:DPPG:Chol (2:5:3 mol ratio), and rigid (iv) DPPC:DPPG (5:5 mol ratio) GUVs in the solid state (So). Since there is no phase diagram for five component membranes (DOPC, DPPC, DOPG, DPPC, and Chol), we use a reported diagram for the three component DOPG:eSM:Chol membranes (48) that also render charged membranes. The position of the tested compositions in the phase diagram is shown in Figure 3*A*. The sketches represent the expected molecular order in these membranes. In addition to the four charged compositions, we also studied their equivalent neutral compositions made of DOPC and DOPC:Chol (7:3 mol ratio), both in the Ld phase, to assess potential contributions of charge on membrane mechanics. All compositions tested here are far from the phase-separated region and thus we expect the membrane to be homogeneous.

**Supporting Figure S2:**
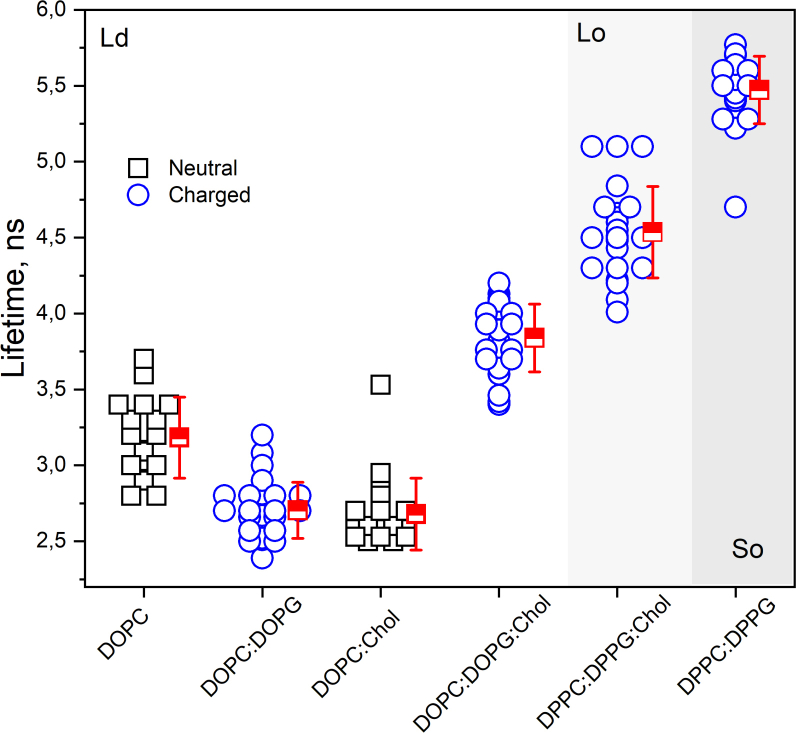
**Measured fluorescence lifetime of the molecular rotor Bodipy C**_**12**_**in neutral (*black squares*) and negatively charged GUVs (*blue circles*) in different phase states (indicated by the *shaded bars*).** The GUVs were labeled with 0.5 mol% of the rotor. Each point represents a measurement on a single GUV. Means and SD are shown.

We first measured membrane viscosity (η), the opposite of fluidity. For this, we used FLIM and molecular rotors as viscosity reporters (49), which have the advantage of revealing the local viscosity (microviscosity) sensed by the probe at optical resolution, even for membranes in the solid state that are typically inaccessible using other methods (50, 51). We used the well-established rotor Bodipy C_12_, a bright probe that sensitively responds to viscosity by changes in fluorescence intensity and lifetime and whose viscosity *versus* lifetime (τ_f_) response is well characterized (52). Representative FLIM images for these four compositions are shown in Figure 3*A*. The lifetime color code from blue to red represents shorter (more fluid) and longer (more viscous) lifetimes, respectively. The fluorescence decays are best fitted with a double-exponential model, from which the longer lifetime was used to assess viscosity. The measured lifetimes for all compositions are shown in Fig. S2. The calculated viscosities for several individual GUVs of the investigated compositions are shown in Figure 3*B*. In general, the inclusion of the charged lipid DOPG or addition of Chol in fluid DOPC membranes causes a mild change in viscosity (300–600 cP). Interestingly, in charged membranes, Chol significantly increases viscosity (900 cP), indicating a synergistic effect to increase fluidity when both molecules are both present. Membranes containing DP lipids and cholesterol (in the Lo phase) are nearly two times more viscous than their DO counterparts in the Ld phase (1200 cP). This shows that compositions rich in fully saturated lipids are more ordered (viscous) despite the identical phase and amount of Chol compared to unsaturated lipids. Not surprisingly, membranes in the So phase are the most viscous of all compositions tested (1800 cP). Thus, we conclude that membrane phase-state, charge, and Chol modulate membrane viscosity, which increase in the following order Ld>Lo>So and that synergistic effects of combined lipids in the membrane have a further effect on viscosity compared to when they are present alone.

**Figure 4:**
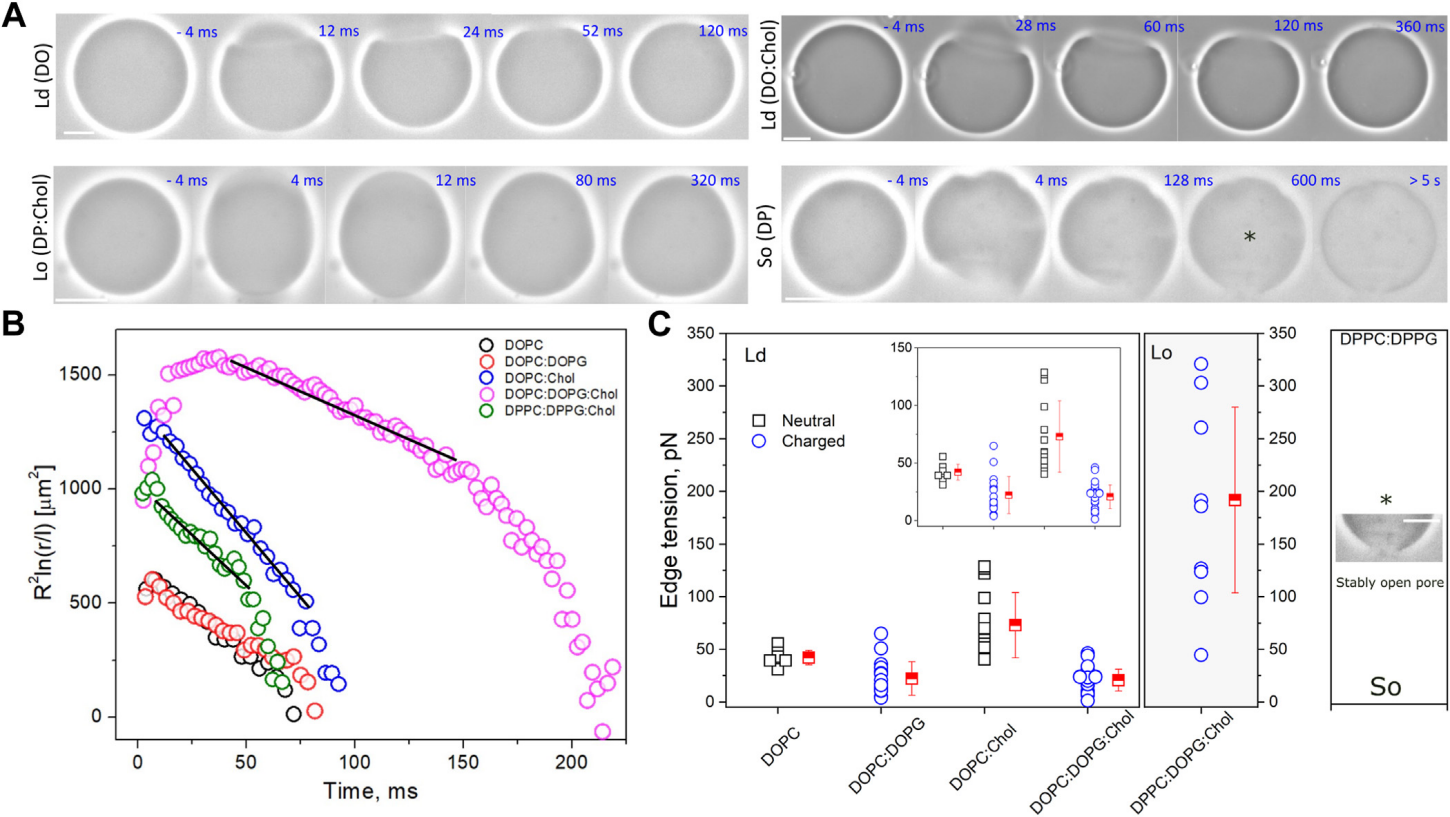
**Edge tension measurements on GUVs of various compositions, charge, and phase-state.***A*, snapshots of representative Ld GUVs of DOPC:DOPG (5:5 mol ratio) and DOPC:DOPG:Chol (2:5:3 mol), Lo GUVs of DPPC:DPPG:Chol (2:5:3 mol ratio), and So GUVs of DPPC:DPPG (5:5 mol ratio). The numbers correspond to time relative to when the pulse was detected (t = 0). Scale bars represent 10 μm. Pulse field strength and duration varied from 3 to 4 kV/cm^−1^ and 150 to 200 μs for DO- and DP-containing membranes, respectively. A respective video for each of the GUVs is shown in Movies S1–S4. *B*, pore closure dynamics for representative GUVs of different compositions. Fits of the slow closure regime are shown (*dashed lines*). *C*, edge tension measurements for several GUVs from the compositions and phase-state tested, for neutral (*black*) and charged (*blue*) membranes. Inset is a zoom-in of Ld GUVs. Each point represents a measurement on a single GUV. Means and SD are shown. Measurements are not possible with GUVs in the So state. A representative “cracked” GUV with an open pore is shown. Note that the image shown as an inset is cropped from the same as in frame 600 ms in Figure A (highlighted as ∗), shown for clarity (bar: 10 μm). DOPC, 1,2-dioleoyl-sn-glycero-3-phosphocholine; DOPG, 1,2-dioleoyl-sn-glycero-3-phospho-(1′'-rac-glycerol); DPPC, 1,2-dipalmitoyl-sn-glycero-3-phosphocholine; DPPG, 1,2-dipalmitoyl-sn-glycero-3-phospho-(1′'-rac-glycerol); GUV, giant unilamellar vesicle; Ld, liquid disordered.

**Supporting Figure S3:**
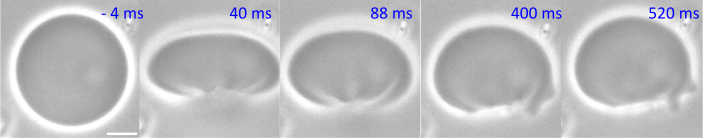
**Wrinkled pores in highly viscous Lo composed of DPPC:DPPG:Chol (2:5:3 mol rato).** Such a morphology was only observed for membrane Lo GUVs (whether neutral or charged) containing Chol. The numbers correspond to time relative to the pulse. Scale bar represents 10 μm.

We next measured membrane edge tension (γ), the energy cost per perimeter of maintaining a pore in the membrane. Its molecular origin arises from bending the lipids away from their preferred configuration to prevent exposure of their hydrophobic tails to water (53). In practice, γ determines membrane stability against pore formation and thus tends to drive pore closure. Given the conical shape of hydrophilic pores, inverse cone-shaped molecules (*i.e.* DOPE, Chol) increase γ, whereas cone-shape ones (*e.g.* detergents) decrease γ^60^. In conditions of reduced γ, membrane stability is compromised and open pores have long lifetimes (53), which may lead to complete pore expansion and vesicle collapse in extreme cases (54, 55). Importantly, the presence of Chol in neutral membranes increases γ^40^, whereas charged lipids have the opposite effect (56). Here, we probed the combined effects of membrane charge, Chol, and phase-states on γ. Edge tension was assessed by following the dynamics of pore closure in GUVs upon electroporation (57). The slow and linear stage of pore closure is fitted with

$$R^{2}\;\ln\left(r\right)=-\frac{2\gamma }{3\pi \eta }t+C$$

where η is the medium viscosity of the external solution (we used 1.133.10^−3^ Pa s), t is time, and C is a time-independent constant (58) according to the theory derived in (59). From the linear fit of R^2^ln(r) during the slow closure regime as a function of time, γ is estimated from the slope of the curve γ =−(3/2)πηa. Figure 4*A* shows representative snapshots of pore closure dynamics in Ld, Lo, and So GUVs. Pores formed upon electroporation are detected as a bright halo in GUV membrane observed under phase contrast microscopy due to the exchange of solutions from the GUV interior and exterior. In more fluid DO-containing membranes (containing Chol or not), pore lifetimes are in the order of ∼100 ms, in agreement with previous results (60, 61). In more viscous Lo GUVs, pore lifetime is shortened to only a few tens of ms, indicating an increase in γ. Interestingly, due to the high viscosity of these membranes, the deformed vesicle shape relaxes much slower (see also slow relaxation of membrane wrinkles in Fig. S3). Electroporation of solid So GUVs leads to membrane cracking and pores that remain indefinitely open (at least minutes), as previously observed (61, 62). Representative electroporation sequences are shown in Movies S1–S4.

Figure 4*B* shows representative pore closure dynamics for each of the fluid GUVs. Neutral vesicles containing Chol exhibit a sharp slope, whereas charged membranes devoid of Chol exhibit a broader slope, reflecting respectively their high and low γ. Measurements of γ for a number of GUVs are shown in Figure 4*C*. The addition of charged DOPG lipids to DOPC membranes reduces γ from 42 ± 7 pN to 22 ± 16 pN, whereas the addition of Chol to these membranes increases γ to 73 ± 31 pN, in agreement with previous studies (40, 56). Interestingly, in DOPC membranes containing both charged lipids and Chol, γ is significantly lower (21 ± 10 pN) than DOPC GUVs alone, indicating that when both molecules are present, charge effects dominate. For the very viscous Lo membranes containing DP lipids, the measured γ is 192 ± 88 pN, on average much higher than for any of the DO membranes studied. These values are comparable to previous measurements in neutral Lo composed of SM:Chol (40). This suggests that for highly viscous DP membranes in the Lo phase, charge has minor effects on γ. Because the measurements are based on pore closure, GUVs in the So state are inaccessible as the formed pores remain indefinitely open. In summary, the results show that the combined effects of membrane charge, Chol, and phase state determine edge tension, with charges being important in fluid membranes, but having negligible effects in highly viscous membranes, which exhibit extremely high γ values. As with membrane viscosity, combinatorial effects of Chol and charged lipids lead to edge tension values that are different from those when both molecules are present separately, indicating the complex nature of lipid interactions on membrane mechanics.

**Figure 5:**
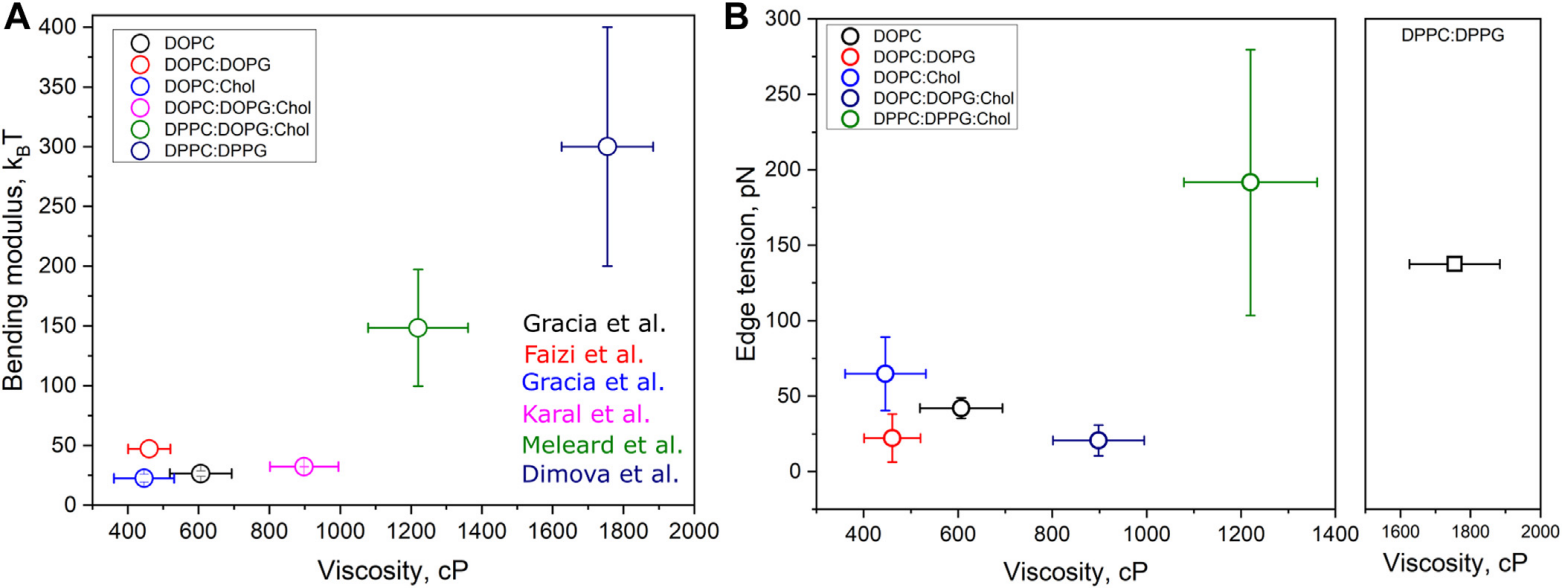
**Correlation between various membrane mechanical parameters.***A*, the dependence of bending modulus on membrane viscosity for membranes of various compositions and phases. For some of the bending rigidity values, the data were obtained from nonidentical (but similar) compositions at the same membrane phase state. For the DOPC:DOPG (5:5) GUVs used here, data from POPC:POPG (50:50) in (66). For the DOPC:DOPG:Chol (2:5:3) GUVs used here, data from DOPC:DOPG:Chol (28:43:29) in (90). For the DPPC:DPPG:Chol (2:5:3) GUVs used here, data from DMPC:Chol (7:3) in (91). For the DPPC:DPPG (5:5) GUVs used here, data from DMPC in the gel state in (92). *B*, dependence of edge tension on membrane viscosity. Note that it is not possible to measure edge tension for membranes in the So phase (DPPC:DPPG), and thus only the viscosity data is shown. Means and SD are shown. DOPC, 1,2-dioleoyl-sn-glycero-3-phosphocholine; DOPG, 1,2-dioleoyl-sn-glycero-3-phospho-(1′'-rac-glycerol); DPPC, 1,2-dipalmitoyl-sn-glycero-3-phosphocholine; DPPG, 1,2-dipalmitoyl-sn-glycero-3-phospho-(1′'-rac-glycerol); GUV, giant unilamellar vesicle; POPC, 1-palmitoyl-2-oleoyl-sn-glycero-3-phosphocholine; POPG, 1-palmitoyl-2-oleoyl-sn-glycero-3-phospho-(10-rac-glycerol).

**Supporting Figure S4:**
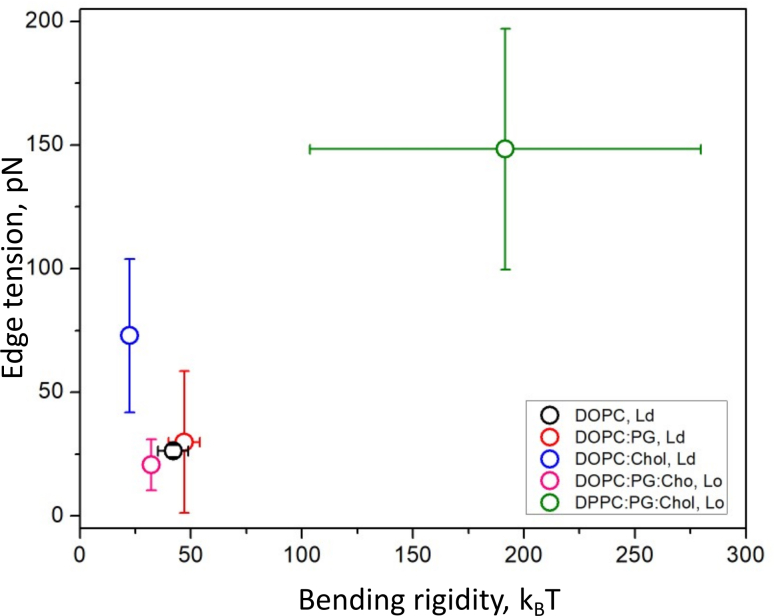
**Correlation between membrane edge tension and bending rigidity for GUVs membranes in different phase states.** Means and SD are shown.

In addition to viscosity and edge tension measurements, we also used bending rigidity (κ) data available in the literature for the relevant membrane compositions used here. Also known as bending stiffness or bending modulus, κ refers to the energy required to bend membranes away from their equilibrium configuration (63). Independent mechanical parameters inform on specific properties of the membrane, but they are often difficult to disentangle given the cooperative nature of lipid–lipid interactions (64). For example, the increase in the fraction of Chol in POPC membranes has been consistently shown to result in membrane stiffening (35, 64), increase in viscosity (34), and in edge tension (40). Hence, we sought to check how much these three parameters are correlated for the relevant membranes used here and as shown below, how they influence the ability of membranes to fuse. Conveniently, measurements of κ have been reported for a broad range of compositions (63, 64, 65), and for those studied here whose measurements are not available, we used data for similar membrane phase states. Figure 5*A* shows that η and κ are strongly and nonlinearly correlated. For very fluid membranes (low viscosity), κ is nearly constant and low, around 25 k_B_T. An exception to this are charged membranes, reported to be stiffer than their neutral counterparts (66). GUVs in the Lo phase containing the fully saturated DP lipids and Chol are both viscous and stiff. Importantly, GUVs in the solid state are extremely stiff and viscous, but for the reasons above, γ is not assessable in these membranes. In contrast, γ does not exhibit a clear correlation with η (Fig. 5*B*) nor with κ (Fig. S4), although the extremely viscous and stiff membranes also exhibit high γ values. As discussed below, we interpret these results as the different molecular origins of these parameters.

### Membrane phase state modulates fusion efficiency and fusion-dependent membrane disruption

**Figure 6:**
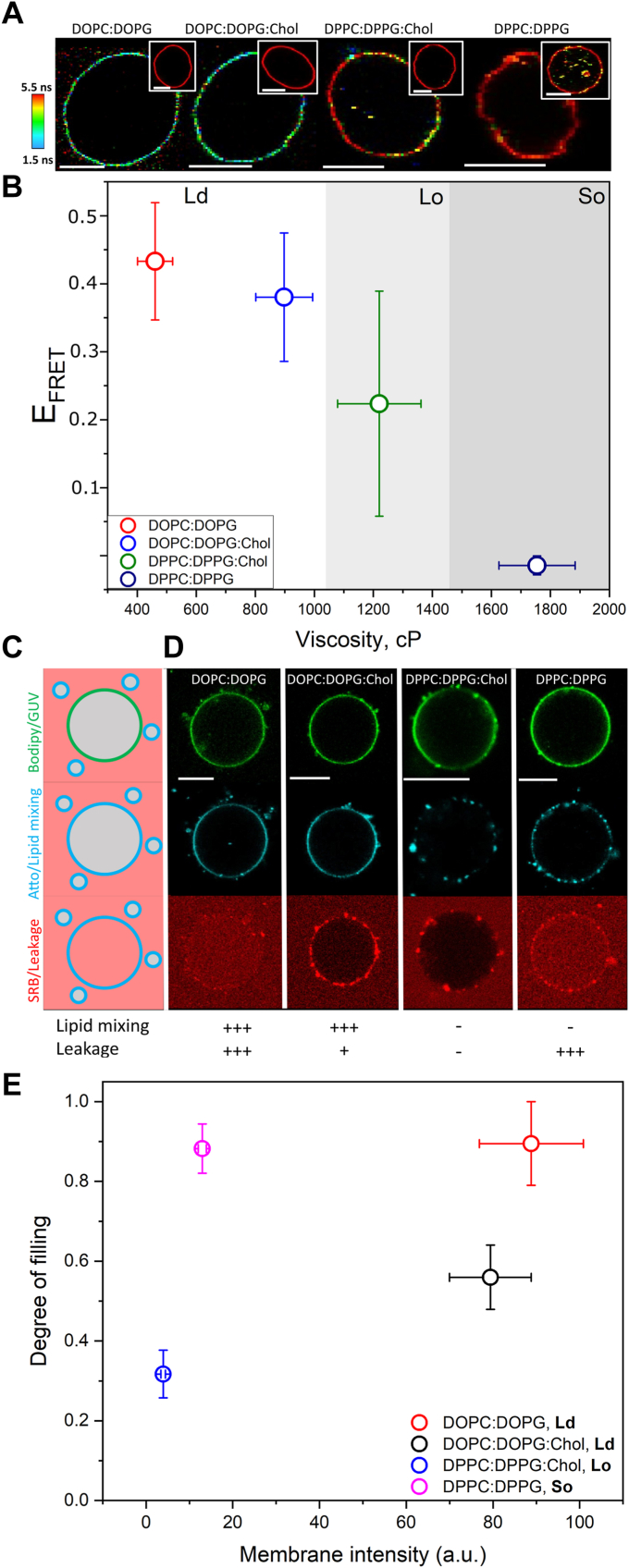
**Fusion efficiency and fusion-dependent disruption inversely depend on membrane viscosity as assessed by FRET-FLIM and a combined fusion/leakage assay.***A*, representative images of the four GUV compositions studied (labeled with 0.5 mol% Bodipy C_16_) upon incubation with 15 μM LUVs labeled with 2 mol% DPPE-Rh. The insets show representative control GUVs (without LUVs). Scale bars represent 9 μm. *B*, dependence of E_FRET_ on membrane viscosity for the GUV compositions studied. Mean and SD (n > 20 GUVs) are shown. The experiments were performed at room temperature (18 ± 1 °C). *C*, sketch of the fusion/leakage assay. Green-labeled GUVs were incubated with cyan-labeled LUVs in the presence of the leakage marker (*red*). The appearance of a cyan signal on the GUV membrane is an indication of membrane fusion. If fusion is leakage free, the leakage marker is retained in the outside medium. In contrast, if fusion is followed by membrane permeabilization, the leakage marker is able to enter the GUV interior. *D*, representative images of GUVs (*green*) incubated with Atto-labeled LUVs (*cyan*). LUVs fuse with fluid Ld GUVs made of DOPC:DOPG (5:5 mol ratio) and DOPC:DOPG:Chol (2:5:3 mol ratio) but not with viscous GUVs in the Lo phase made of DPPC:DPPG:Chol (2:5:3 mol ratio) or So GUVs made of DPPC:DPPG (5:5 mol ratio). The experiments were performed on the presence of SRB as a leakage marker. Intact GUVs exhibit a dark interior whereas permeabilization allows SRB entry. The GUVs were labeled with 0.5 mol% Bodipy C_16_. LUV concentration: 25 μM LUVs (lipid concentration). SRB concentration: 10 μM. Scale bar represents 10 μm. *E*, dependence of the degree of filling on membrane fusion for the GUV compositions studied. DOPC:DOPG (50:50 mol ratio) and DOPC:DOPG:Chol (20:50:30 mol ratio) are membranes in the Ld phase. DPPC:DPPG:Chol (20:50:30 mol ratio) are membranes in the Lo state. DPPC:DPPG (50:50 mol ratio) are membranes in the So state. Means and SEM are shown. DOPC, 1,2-dioleoyl-sn-glycero-3-phosphocholine; DOPG, 1,2-dioleoyl-sn-glycero-3-phospho-(1′'-rac-glycerol); DPPC, 1,2-dipalmitoyl-sn-glycero-3-phosphocholine; DPPE-Rh, 1,2-dipalmitoyl-sn-glycero-3-phosphoethanolamine-N-(lissamine rhodamine B sulfonyl); DPPG, 1,2-dipalmitoyl-sn-glycero-3-phospho-(1′'-rac-glycerol); FLIM, fluorescence lifetime imaging microscopy; GUV, giant unilamellar vesicle; Ld, liquid disordered; LUV, large unilamellar vesicle; SRB, sulforhodamine B.

**Supporting Figure S5:**
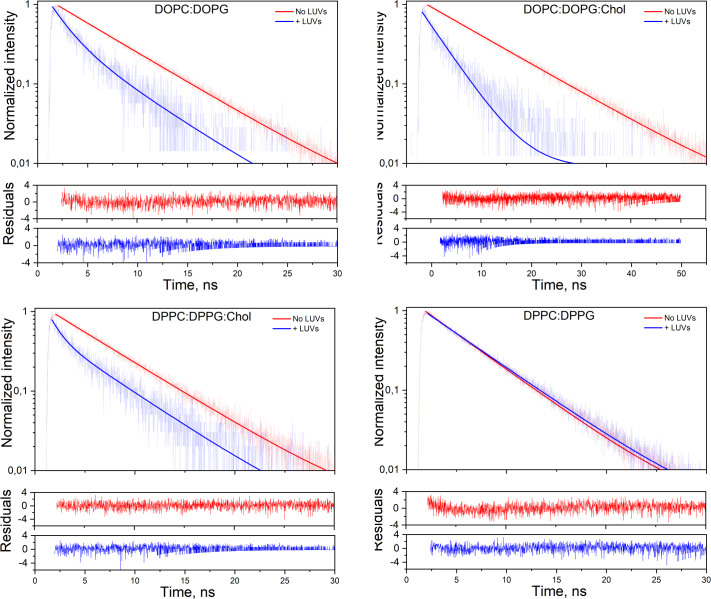
**Representative fluorescence decays for the GUVs in the different membrane phases in the absence (control, *red*) and presence of 15 μM LUVs (*blue*).** The data fits and respective residuals are also shown.

**Supporting Figure S6:**
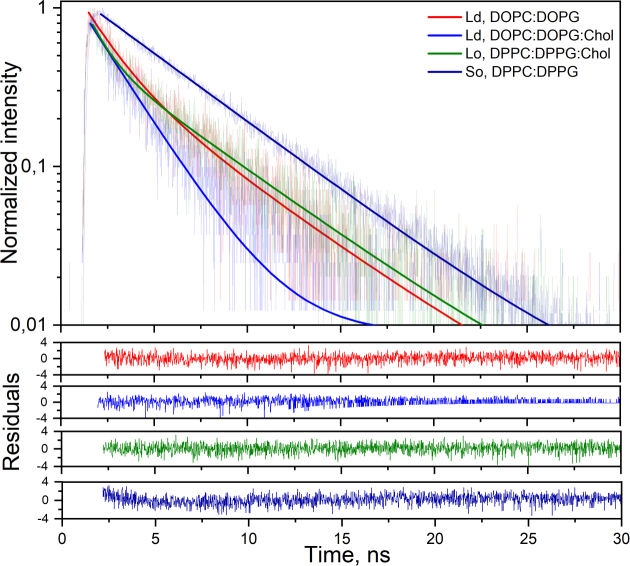
**Representative fluorescence decays for the GUVs in the different membrane phases in the presence of 15 μM LUVs.** The data fits and respective residuals are also shown.

**Supporting Figure S7:**
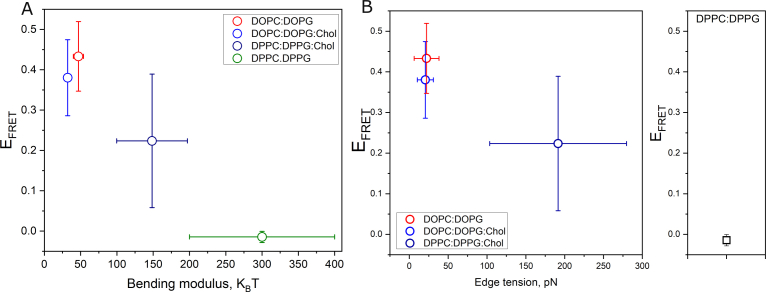
**Correlation between the degree of filling upon fusion of LUVs with GUVs in different membrane phases with membrane edge tension.** Means and SD are shown.

Having systematically characterized the effects of lipid composition on the mechanics of membranes of different phases, we next studied how the LUVs fuse with these mechanically very diverse GUVs. Since the fusion driver in our system is of electrostatic origin, all membranes contained the same amount of charge (equal mol fraction of anionic lipids). FLIM-FRET was used to determine fusion efficiency of fusogenic LUVs with GUVs. In this assay, only the signal from the donor dye is detected, and therefore the assay does not suffer interference from docked LUVs (labeled with the acceptor). Figure 6*A* shows representative FLIM images of the GUV compositions tested upon incubation with the LUVs. The fluorescence decays and their fits are shown in Figs. S5 and S6. The quantification of E_FRET_ for several GUVs (n ∼20 per composition) shows that fusion efficiency progressively decreases depending on membrane phase state, following the order: Ld>Lo>So. In fact, when we plot E_FRET_ as a function of viscosity, there is a clear inverse trend (Fig. 6*B*), until no fusion is observed with highly viscous So GUVs. This effect is also mirrored by increases in bending rigidity (Fig. S7*A*) and less obvious with edge tension (Fig. S7*B*).

To study potential side effects of fusion on membrane disruption, we tested whether fusion is a leakage-free process or if it instead leads to the formation of leakage pores in the membranes. This is relevant because it is often assumed that in cells, fusion proceeds without pore formation outside the fusion area (13, 19, 21), an effect that has been disputed (41, 42, 43), as well as for its relevance in fusion-based drug-delivery systems. To simultaneously assess fusion and membrane disruption, we used multicolor confocal microscopy, in which the GUVs are identified based on their green labeling, fusion is detected as the lipid (DOPE-Atto) transfer from the LUVs (lipid mixing, cyan) to the GUV membrane, and membrane disruption is detected by the entry of SRB (red) in the GUVs. SRB is a small water-soluble probe that is unable to transverse intact membranes and is thus retained outside. However, if pores are present, SRB can enter the GUVs at an extent that depends on the level of disruption (Fig. 6*C*).

**Supporting Figure S8:**
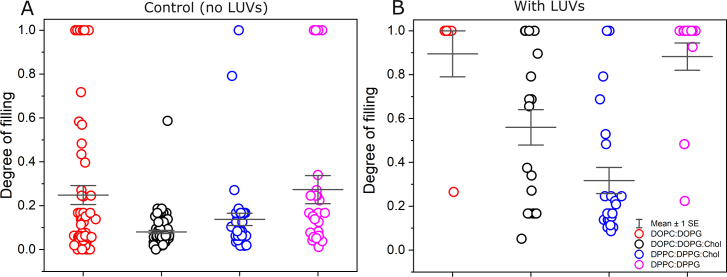
**Single vesicle fusion-dependent permeability.***A*, control GUVs in the absence of LUVs. Note that there is some background permeability. *B*, degree of filling for GUVs in the presence of 15 μM LUVs (lipid concentration). Permeabilization is graded for GUVs with intermediate viscosities and all-or-none for very fluid membranes. Each circle represents a measurement on an individual GUV. The bars represent the mean and SEM.

**Supporting Figure S9:**
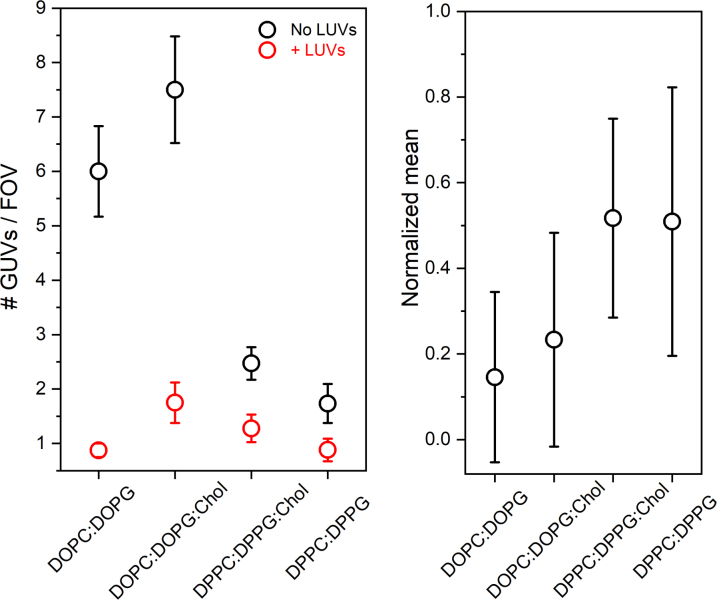
**LUV fusion induces disruption that depends on membrane phase state.***A*, the number of GUVs per field of view measured for control (no LUVs) and after incubation with 25 μM LUVs. In (*B*), the normalized number of GUVs per field of view shows that more GUVs remain upon LUV incubation with more viscous GUVs. Means and SEM are shown.

**Supporting Figure S10:**
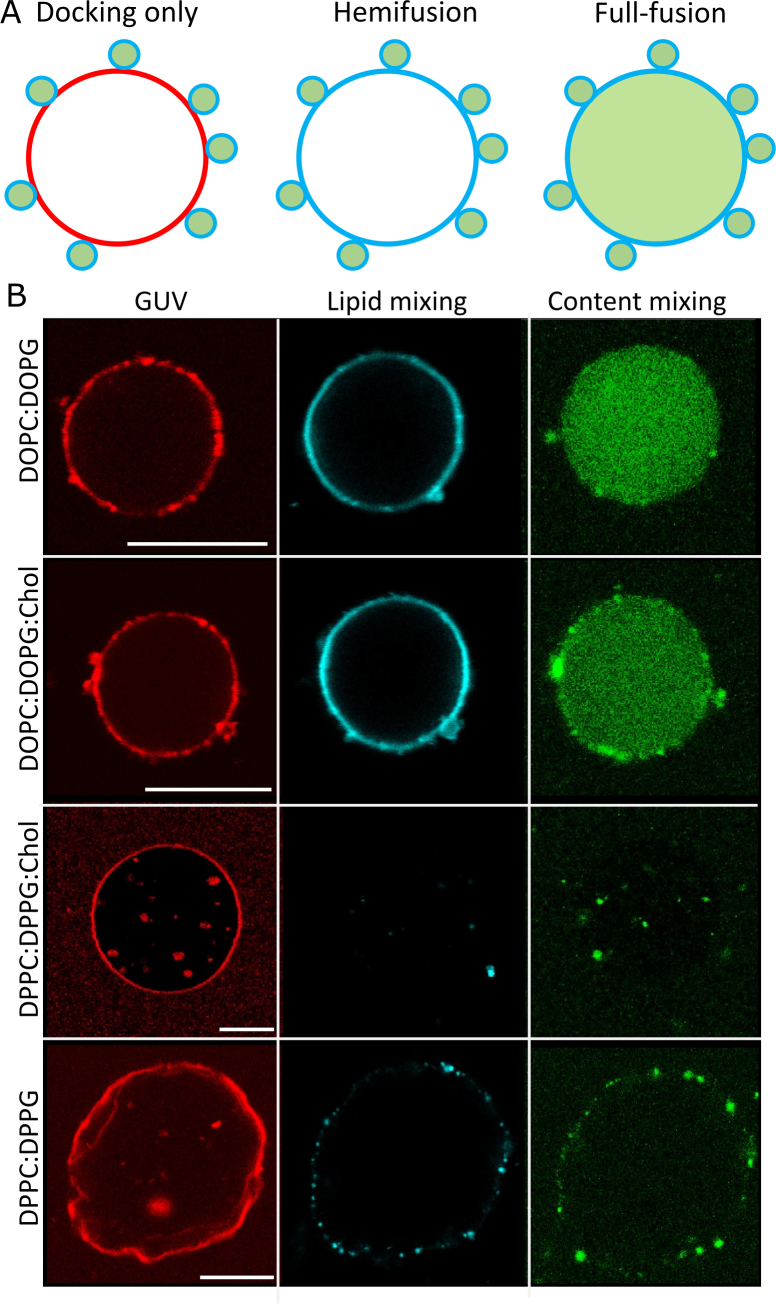
**Content mixing assay with GUVs of different phases.***A*, sketch of the fusion assay. The red labeled GUVs (labeled 0.5 mol% DPPE-Rh) were incubated with the fusogenic LUVs labeled at the membrane with 0.5 mol% Atto (cyan) as a lipid mixing marker and encapsulating Dextran-FITC 3 kDa (0.1 mg/ml) as a content marker. If the LUVs only docked to the GUVs, diffraction limited spots are seen in the lipid (*cyan*) and content mixing channel (*green*). If only hemifusion takes place, hemifused LUVs will be seeing at the GUV membrane, which also becomes *cyan*. If full-fusion takes place, both the lipid marker (*cyan*) as well as the content marker (*green*) are observed, at the GUV membrane and internal compartments, respectively. *B*, LUVs efficiently fuse and undergo content mixing with fluid GUVs, as seen by efficient lipid (*cyan*) and content mixing (*green*). The LUVs dock (seeing as diffraction-limited spots in both the lipid and content channels, *arrows*) but do no undergo extensive lipid mixing with viscous GUVs. There is no detectable transfer of the content marker. Note that the GUVs that had undergone efficient fusion also exhibit outward buds in their membranes (arrowheads), a sign of lipid transfer (*i.e. via* fusion) from the LUVs. LUV (lipid) concentration: 25 μM (total lipid). Scale bar represents 10 μm.

**Supporting Figure S11:**
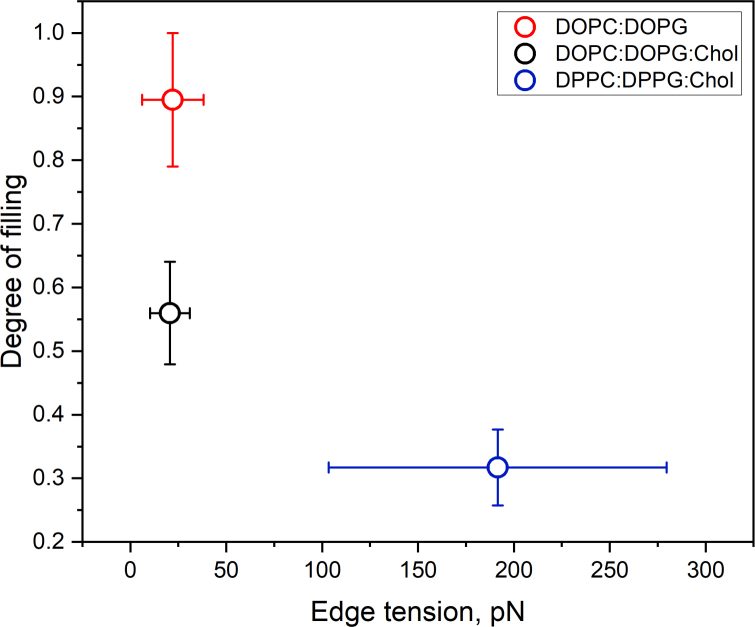
**Membrane permeabilization (assessed by the degree of filling) does not have a clear correlation with edge tension.** Means and SD are shown.

The reasoning behind these experiments is that more rigid membranes may be less fusion-prone, based on the FLIM-FRET experiments above, but also more mechanically stable given by their high edge tension. Figure 6, *C*–*E* show that the fluid Ld GUVs are very permissive to fusion, exhibiting high membrane signal from transferred Atto from the LUVs (*i.e.* fusion), with Chol-containing GUVs undergoing slightly less fusion. At the same time, these membranes also become permeable to SRB, with Chol-containing GUVs being more resistant against pore formation (lower DOF). Single GUV analysis shows that permeabilization is an all-or-none process for GUVs without Chol and graded for Chol-containing GUVs (Fig. S8), pointing out to a role of Chol as a mechanical stabilizer. Because in our system extensive fusion is a disruptive process as it permeabilizes the membrane and reduces the total number of GUVs (Fig. S9), it is not clear whether the lower permeabilization in Chol-containing GUVs is due to a lower extent of fusion or due to specific Chol effects (see below). Unlike very fluid membranes, the LUVs extensively dock on very viscous membranes, regardless of the membrane phase (Lo for DP-Chol or So for DP-only GUVs), but they do not seem to fuse–the presence of black gaps in the images points to docked rather than fused LUVs. In fact, while the fluid membranes undergo content-mixing, the viscous membranes do not (Fig. S10). This demonstrates that very viscous membranes are less permissive to fusion, in agreement with Cavalcanti *et al.* (32). The very viscous DP membranes containing Chol are also very resistant to permeabilization, even though no clear correlation between the extent of membrane permeabilization and edge tension is observed (Fig. S11); having only three data points (*i.e.* membrane compositions) preclude more solid conclusions. The So GUVs also exhibit a high DOF as defects formed in these membranes during preparation are not able to reseal due to their nonfluid nature but this effect is independent of fusion.

We conclude that fusion occurs very efficiently with fluid membranes, it proceeds *via* full fusion, but it becomes progressively inefficient as membrane viscosity and stiffness increase. As the membranes become more viscous, they also seem to become more mechanically resilient and able to withstand fusion without disruption.

### Fusion becomes increasingly more efficient as membranes fluidize upon temperature shifts

**Figure 7:**
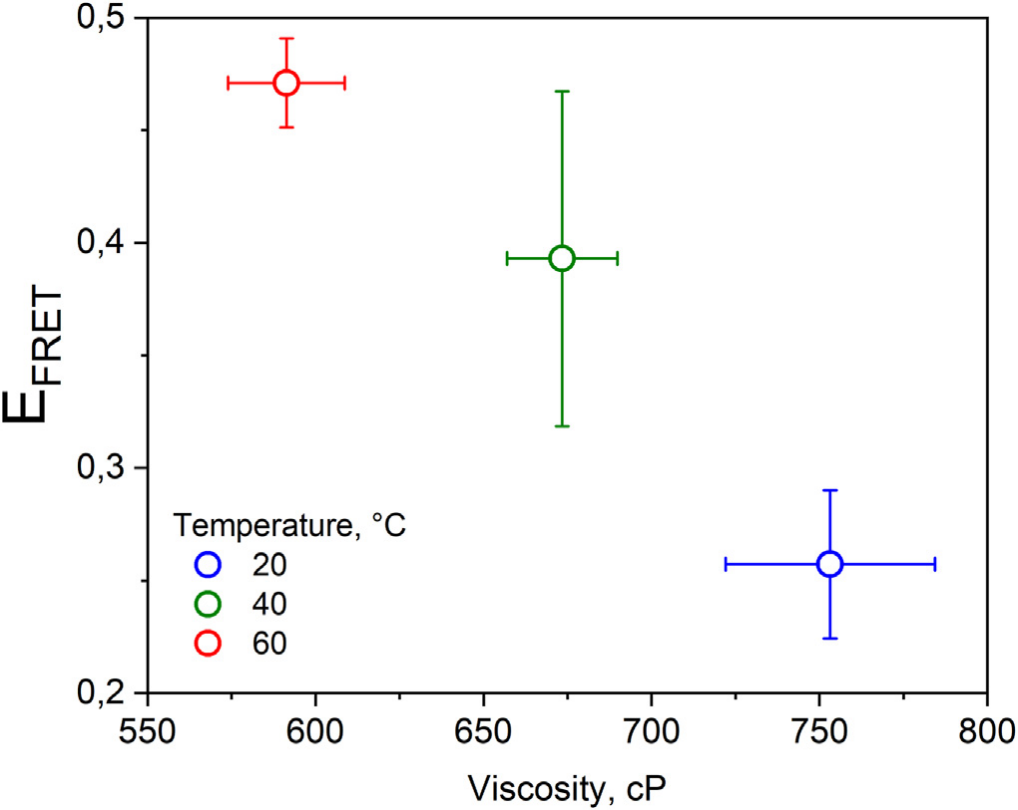
**Fusion inversely scales with viscosity as assessed upon temperature shifts.** Lo GUVs made of DPPC:DPPG:Chol (2:5:3 mol ratio) were labeled with 0.5 mol% of either Bodipy C_12_ or Bodipy C_16_ for the fluidity and FRET experiments, respectively. In the fusion experiments, the GUVs were incubated with 15 μM LUVs (total lipids) labeled with 2 mol% DPPE-Rh. The data display the mean and SEMs for viscosity and E_FRET_. DPPC, 1,2-dipalmitoyl-sn-glycero-3-phosphocholine; DPPE-Rh, 1,2-dipalmitoyl-sn-glycero-3-phosphoethanolamine-N-(lissamine rhodamine B sulfonyl); DPPG, 1,2-dipalmitoyl-sn-glycero-3-phospho-(1′'-rac-glycerol); GUV, giant unilamellar vesicle; LUV, large unilamellar vesicle.

**Supporting Figure S12:**
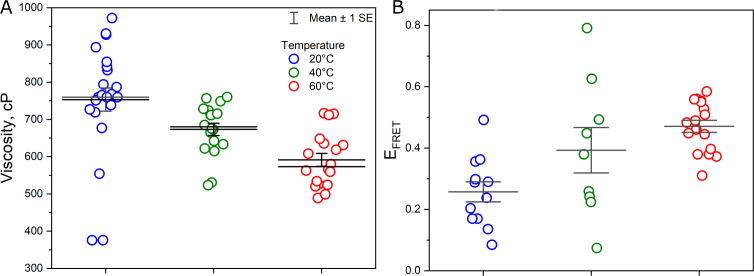
**Single GUV data for DPPC:DPPG:Chol GUVs (2:5:3, mol ratio).***A*, temperature-dependence on membrane viscosity. The GUVs were labeled with 0.5 mol% Bodipy C_12_. *B*, temperature-dependence on E_FRET_. The GUVs were labeled with 0.5 mol% Bodipy C_12_ and incubated with 15 μM LUVs (total lipids) and labeled with 2 mol% DPPE-Rh.

In the experiments above, we show that the underlying mechanical properties tune the ability of membranes to fuse, and higher viscosity/bending rigidity translate into lower fusion efficiency. Since the experiments above were carried out with membranes of different compositions and phase state and since it was not possible to subtly modify membrane viscosity isothermally with Chol (see below), we then tuned the viscosity of GUV membranes of a fixed composition by temperature shifts. For this, we chose Lo GUVs made of DPPC:DPPG:Chol, which at room temperature are very viscous and whose fusion efficiency is low, and we used FLIM-FRET to avoid interference from LUV docking. As can be seen in Figure 7, at 20 °C, close to R.T., E_FRET_ is low but detectable. As the temperature increases, the membrane becomes progressively more fluid (observed as a reduction in viscosity). Importantly, fusion also becomes progressively more efficient, in agreement with the data for membranes in different phase states. Single vesicle viscosity and E_FRET_, which are independently measured, are shown in Fig. S12. We also performed similar measurements with DPPC:DPPG GUVs below and above the membrane Tm. However, the quality of the GUVs above Tm and upon incubation with the LUVs was very poor and therefore we could not analyze the data. We conclude that the fusion dependence on mechanical properties is likely a more general effect that does not depend on the specific lipid composition.

### Cholesterol increases the mechanical resilience of membranes

**Figure 8:**
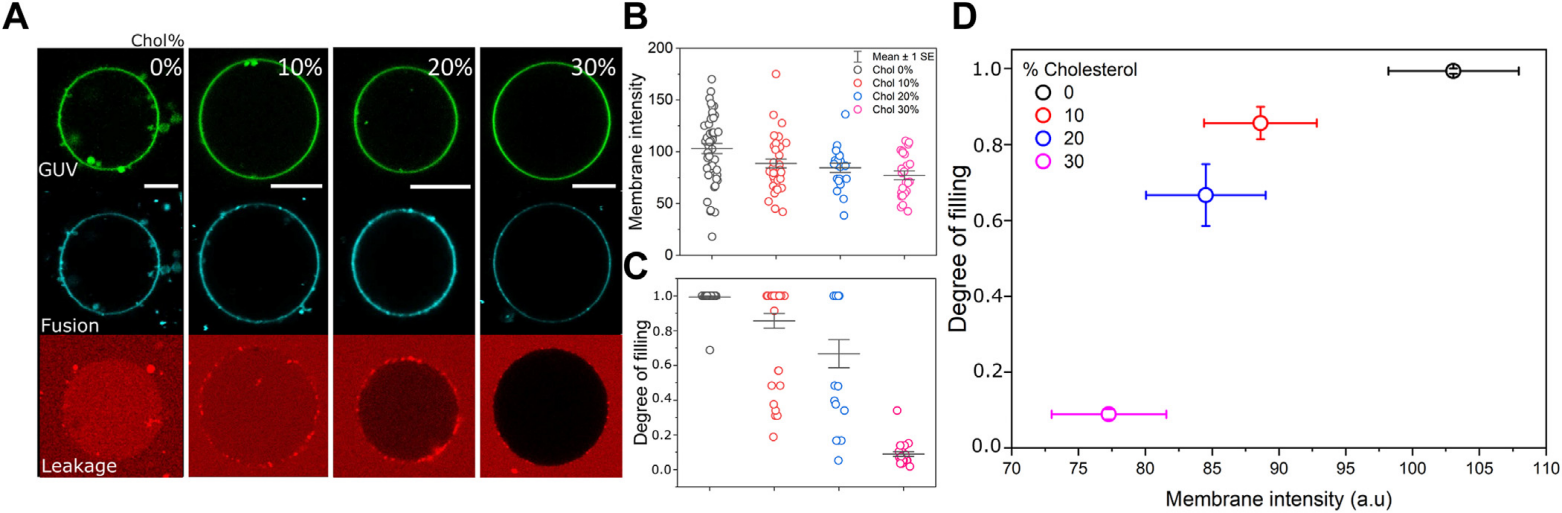
**Cholesterol reduces the ability of membranes to fuse and increases mechanical resilience against pore formation.***A*, representative GUVs (POPC:PS:Chol, X:50:50-X, mol ratio) containing increasing fractions of Chol (X) at the expense of POPC. The GUVs (*green*) were labeled with 0.5 mol% Bodipy C_12_ and incubated with 15 μM (total lipids) Atto-labeled LUVs (*cyan*) in the presence of 10 μM SRB as a leakage marker. Fusion efficiency was assessed by measuring the transfer of Atto from the LUVs to the GUVs. Scale bar represents 10 μm. *B* and *C*, measured Atto intensity transferred from the LUVs *via* membrane fusion (lipid mixing) and measurements of the degree of filling as a proxy for membrane disruption, respectively, for increasing fractions of Chol. Each point represents the measurements on an individual GUV. Mean and SEM are also shown. *D*, dependence on the degree of filling on membrane fusion intensity (lipid mixing) for increasing Chol mol%. GUV, giant unilamellar vesicle; LUV, large unilamellar vesicle; POPC, 1-palmitoyl-2-oleoyl-sn-glycero-3-phosphocholine; PS, phosphatidylserine; SRB, sulforhodamine B.

**Supporting Figure S13:**
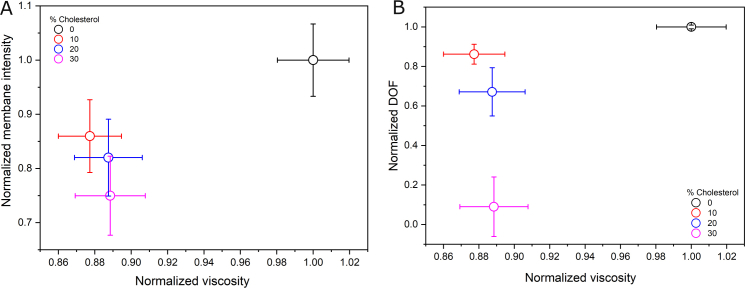
**Chol has negligible effects on viscosity, reduces fusion efficiency, and stabilizes membranes against fusion-mediated pore formation.** LUV concentration: 15 μM (lipids). Viscosity was assessed from the lifetime of Bodipy C_12_ (0.5 mol%).

The experiments above indicate that the underlying mechanics for very different membranes tune their ability to fuse as well as on the downstream effects of membrane disruption (*i.e.* disruption pores). To address these matter in more detail, here we sought to increase membrane viscosity more subtly by adding Chol to the GUVs. Because Chol interacts poorly with lipids that exhibit a higher degree of unsaturation (63, 65), we use POPC as the zwitterionic lipid and brain PS, rich in monounsaturated lipids, as the anionic lipid—instead of DOPC and DOPG, respectively—due to the higher saturation degree of these lipids. The GUVs produced using these lipids are still in the Ld as expected based on the low Tm of their constituent lipids and as judged by membrane fluctuations (not shown). We used the multicolor confocal assay to simultaneously assess fusion from Atto transferred from the LUVs and SRB leakage through the membrane to assess disruption. Figure 8*A* shows the fusion channel (cyan) and the permeability channel (red)—the GUVs are identified by their initial green color. To our surprise, Chol does not increase the viscosity in these membranes nor is fusion or fusion-dependent disruption dependent on viscosity (Fig. S13). However, incremental additions of Chol lead to a mild and progressive reduction of fusion (Fig. 8*B*) as well as a significant decrease in the membrane permeabilization. For membranes devoid of Chol, permeabilization follows an all-or-none mechanism, whereas the addition of mild fractions of Chol changes it to a graded mechanism (Fig. 8*C*), hence less severe disruption effects. With 30 mol% Chol, the GUVs are no longer permeable upon fusion. Figure 8*D* shows the correlation between membrane fusion and permeabilization as a function of Chol fraction. Of note, for GUVs containing 10 to 20 mol% of Chol, the fusion efficiency is identical within error whereas the degree of filling is significantly reduced with higher Chol fractions. These findings confirm that Chol increases membrane resilience against pore formation for identical extents of fusion.

## Discussion

Our work sheds new light onto the effects of membrane mechanics and fusion. The study provides values for membrane viscosity and edge tension and compares these to reported literature data for bending rigidity. Membrane fluidity is analogous to liquid fluidity where it refers to the ability of molecules to move in a fluid and the corresponding fluid resistance to deformation (67). Bending rigidity is a measure of membrane resistance against deformation from their equilibrium shape (63, 65). Edge tension refers to the energy associated of having a defect (*i.e.* a hydrophilic pore) in the membrane as a function of defect length (52, 58). All these properties have been separately shown to be critically important in a multitude of biological processes, from signaling (68) and cellular migration (69) to homeoviscous adaptation in prokaryotes (70) and cellular repair upon membrane damage (71). Strikingly, they are commonly referred to as important factors in membrane fusion, although systematic characterization of their effects on fusion is largely lacking.

What these three (and other) mechanical factors have in common is their dependence on intrinsic (*i.e.* composition) and extrinsic (*i.e.* temperature) factors. Here we show that membrane viscosity, bending rigidity, and edge tension are separately modulated by membrane charge and Chol and that charge and Chol together interact to further modulate these properties. In general, Chol alone increases viscosity whereas charge alone has milder effects, and viscosity is significantly higher in membranes containing saturated lipids, regardless of the presence of Chol or charge. These effects are mirrored in bending rigidity of membranes. In contrast, membrane edge tension is strongly dependent on charges and Chol separately. Adding charges decreases membrane edge tension, as previously reported (56), whereas Chol has the opposite effect, also in agreement with previous data (40). However, when combined, membrane edge tension is similar to that in charged membranes when Chol is present, and it does not correlate well with changes in viscosity or bending rigidity.

The reasoning behind this weak dependence is their molecular origin. The viscosity as measured here corresponds to the local environment immediately around the molecular probe, the microviscosity. It assesses local membrane packing, mainly defined by hydrophobic interactions of the phospholipid tail regions, effectively reducing the rotational degree of freedom of the rotor probe. On the contrary, edge tension is a more cooperative and larger scale parameter and assesses the energy required to maintain a large defect in the membrane, whose origin derives from the lipid molecular geometry. In other words, it is ultimately defined by the area ratio between the headgroup and tail regions of the lipid molecules. Thus, viscosity and edge tension have different origins that are not exactly directly connected. Whereas the former depends on the degree of lipid–lipid interactions that determines membrane packing, the latter is related to the particular lipid geometry that match the positive curvature of the pore. In fact, for the compositions measured, the bending modulus, which strongly correlates with membrane viscosity, also does not show a clear correlation with edge tension. Hence, the different mechanical properties of membranes may be directly linked, but that degree of correlation likely depends on their molecular origin.

We show that by modifying these mechanical properties, the ability of membranes to fuse varies universally over the tested parameter space. Analysis of several mechanical parameters show that such effects are rather general provided other factors such as charge are kept constant and thus not strictly dependent on membrane composition *per se*. This has been confirmed by altering the lipid composition, the degree of lipid saturation, membrane phase state, temperature, and cholesterol fraction. The findings experimentally confirm molecular dynamics simulations in which the more fluid inner leaflet of the PM is more fusogenic than their more viscous and ordered outer counterpart (27). We hypothesize that the high charge density promotes strong LUV–GUV interactions, similar to ligand–receptor interactions in biological fusion, but these interactions can only lead to fusion if the membranes are sufficiently fluid to allow membrane remodeling. Otherwise, bending the membranes towards highly curved fusion intermediates becomes extremely costly and hence unfavorable. This explains why very viscous GUVs have stably docked LUVs on their surface that fail to fuse.

Fusion also results in membrane permeabilization and leakage of water-soluble contents. This effect is partially or fully reversed by Chol, and Chol alone has a mild but detectable effect on reducing fusion efficiency. The effect of Chol on fusion contrasts with many reports in the literature. While Chol has an effect to increase bending rigidity, this effect is not universal but instead depends on the specific lipid composition (65). In fact, for the membranes used here, we did not measurably detect changes in viscosity upon addition of Chol. Instead, we hypothesize that the effects of Chol on promoting fusion is more indirect, most likely by providing a favorable environment for fusion proteins. In protein-mediated membrane fusion, Chol promotes the optimal localization of protein domains in the membrane (43, 72); it increases the opening of fusion pores, an effect that seems to depend on protein clustering (73). Chol also increases membrane docking (74); it obviates fusion intermediates, bypassing hemifusion, and increasing fusion speed (29, 30, 75) and modulates the line tension of membrane domains (29, 30), favoring fusion due to the presence of membrane defects, although Chol-induced increase in membrane rigidity has also been suggested as imparting fusion due to the high energy required for membrane remodeling (76).

**Figure 9:**
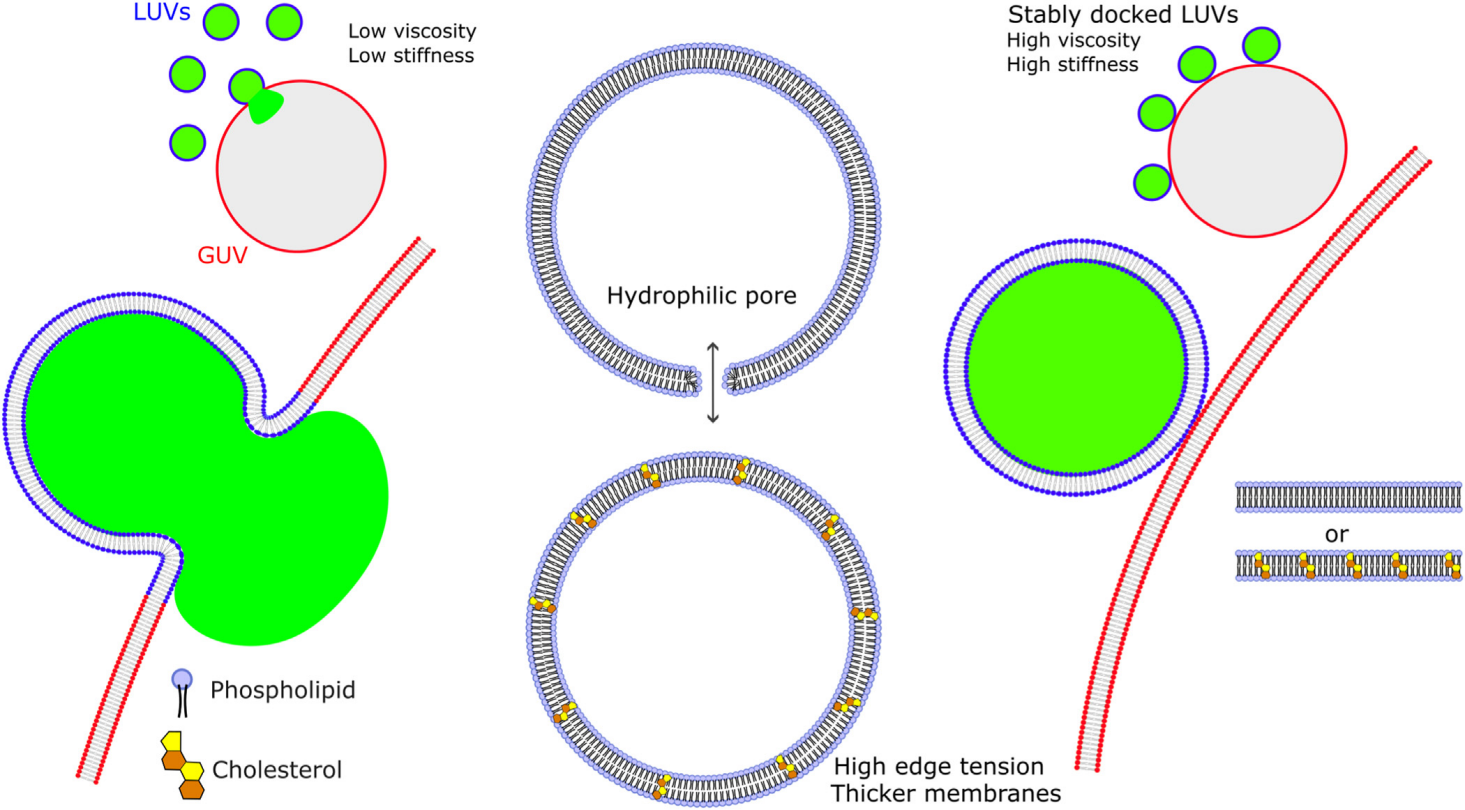
**General effects of membrane mechanics on fusion.** The sketch illustrate LUVs labeled at the membrane (*blue*) and encapsulating a water-soluble marker (*green*) in contact with fluid or viscous GUVs (*red*), containing or not Chol. Highly fluid membranes exhibiting low viscosity (η) and low bending rigidity (κ) are permissive to fusion. In these membranes, fusion proceeds *via* full-fusion, culminating with the complete mixing of the lipid and content of the fusing membranes. As a result, if these fused membranes do not contain cholesterol, fusion induces the formation of hydrophilic pores through which encapsulated aqueous-soluble molecules can escape. In contrast, the presence of Chol in the fused membranes prevents pore formation, preventing content leakage. For very stiff GUVs that exhibit high κ and η, the LUVs stably dock but are unable to fuse. Stable docking does not result in pore formation. Cholesterol has no effect on fusion or permeabilization in these GUVs. GUV, giant unilamellar vesicle; LUV, large unilamellar vesicle.

On the other hand, Chol significantly increases membrane stability against pore formation. Fusion-dependent membrane leakage has been consistently observed in several different circumstances (77). The coincidence of leakage with the early stages of fusion indicates disruption during membrane rearrangements through nonbilayer structures (78). Although the molecular mechanisms of fusion-dependent membrane permeabilization may differ, membrane rupture seems to occur outside the fusion pore (43). Therein, Chol stabilizes membranes against permeabilization due to its negative spontaneous curvature (42) and thus edge tension, but also by increasing membrane thickness (41), especially at the leakage point. The latter reduces lipid rarefactions formed upon an increase in membrane tension (*i.e.* upon mechanical action of fusion proteins) and water penetrability (79). Altogether, these effects decrease the likelihood of pore formation (*i.e.* high membrane thickness) and closure of formed pores (*i.e.* edge tension). A summary of the effects of membrane mechanics on fusion is shown in Figure 9. Of note, although we have previously reported fusion of cationic liposomes as leakage-free (46), here we show that this is only true for mild liposomal concentrations. At higher concentrations, their fusion induces disruption of GUV membranes, firstly by the formation of leakage pores, and at much higher concentrations, complete vesicle collapse occurs. In either case, Chol increases mechanical robustness.

Of note, we take advantage of the outstanding ability of the cationic fusogenic LUVs to fuse with negative membranes (46), a property that is unique to these liposomes when using reconstituted approaches compared to other fusing systems, including those containing SNARE proteins. For this reason, potential changes in the factors related to fusion (*i.e.* its dependence on viscosity or its effects on disrupting membranes) are more likely to be sensitively observed in the regime of high fusion (*i.e.* when many LUVs fuse to a single GUV) than at lower fusion. Indeed, some of us have used SNARE-reconstituted membranes made of lipids or polymers (or mixtures thereof), with indications that fusion is favored when the membranes are more flexible (Otrin, Nature Comm. 12, 2021). Furthermore, we have recently addressed the effects of membrane heterogeneity on fusion using phase-separated GUVs (Cavalcanti *et al.*, Biophys. J. 122, 2023), with heterogeneous membranes being more permissive to fusion, mirroring the effects found in SNARE fusion (29, 30, 31). Lastly, we anticipate future studies to address membrane compositional asymmetry as a new degree of membrane organization and its effects on fusion.

We must emphasize that, although we believe that the biophysical outcomes are likely general in the fusion of small to quasi-flat membranes of the GUVs, these effects were observed in the fusion of protein-free membranes. For reactions mediated by fusion proteins, fusion is additionally regulated by more complex lipid–protein interactions, such as PIP_2_-dependent SNARE multimerization (80), receptor clustering (81), ligand-receptor density (21), hydrophobic mismatch (82), lipid-protein electrostatics (83, 84), to name a few. Furthermore, the study is limited to homogeneous membranes. Whereas some fusion proteins induce more efficient fusion in phase-separated membranes (29, 30, 31), such effects have also been observed in protein-free fusion (32), and this again highlights the active role played by lipids in the fusion reaction. Although fusion *in vivo* is tightly regulated by protein factors, a major role seems to be overcoming of energy barriers associated with the fusion steps, including bending, curving, and rupturing the membrane, all of which are determined by the underlying membrane mechanics we studied. Hence, it is conceivable that specific lipid and protein effects come together with more general properties so that the local composition of the fusing membranes is defined as an interplay between efficient protein activity and efficient fusion while preserving membrane resistance to pore formation. This may also explain why Chol is present at such a high fraction in the PM.

## Conclusions

Membrane fusion depends on the underlying mechanics of the membrane. Although this has been generally known, the precise dependence of fusion on mechanics remained elusive. In this work, we used FLIM and electroporation to assess key mechanical parameters of membranes, such as viscosity and edge tension, respectively, and correlated them with bending rigidity. Furthermore, FLIM-FRET and multicolor confocal microscopy were used to resolve fusion intermediates (docking, lipid, and content mixing as well as membrane leakage). We systematically varied membrane mechanics by changing membrane charge, the level of lipid saturation, phase state, temperature, and Chol levels and demonstrate that fusion efficiency depends more universally on the underlying membrane mechanics, not on specific compositions. Under conditions of strong interaction, fusion is promoted if the membranes are fluid, whereas it becomes progressively impaired as membrane viscosity/bending modulus increases. This is presumably due to the high energy associated with membrane remodeling as required for the transitions through the fusion intermediates. Whereas it has been hypothesized that Chol favors fusion, the results shown here reveal that in protein-free membranes, Chol mildly reduces fusion efficiency while it significantly increases membrane resistance against fusion-dependent pore formation. We believe that the results provide an important understanding of the molecular mechanisms of membrane fusion as well as on its consequences on membrane stability, both in cells as with fusion-based drug delivery carriers, and reconcile literature data on the specific effects of lipid composition and its effects on membrane mechanics that ultimately tune fusion. Whereas in general, leakage is an undesirable outcome of fusion, which may lead to the quick and complete depletion of small vesicular content, in some cases, controlled leakage can be advantageous, wherein regulated release of specific molecules or genetic material is required (85). The mechanical rather than the compositional dependence on fusion offers the cells a much higher degree of freedom to choose the chemical identity in their lipid repertoire so that the membrane is simultaneously permissive to fusion and mechanically stable, leading to evolutionary advantages of the cell.

## Experimental procedures

All materials and chemicals were used as obtained without further purification. The phospholipids 1,2-dioleoyl-sn-glycero-3-phosphocholine (DOPC), 1,2-dioleoyl-sn-glycero-3-phospho-(1′-rac-glycerol) (sodium salt) (DOPG), 1-palmitoyl-2-oleoyl-sn-glycero-3-phosphocholine (POPC), 1-palmitoyl-2-oleoyl-sn-glycero-3-phospho-(10-rac-glycerol) (sodium salt), 1,2-dioleoyl-sn-glycero-3-phosphoethanolamine (DOPE), and 1,2-dioleoyl-3-trimethylammonium-propane (DOTAP) L-α-PS (Brain, Porcine) (sodium salt); 1,2-dipalmitoyl-sn-glycero-3-phosphocholine (DPPC), 1,2-dipalmitoyl-sn-glycero-3-phospho-(1′-rac-glycerol) (sodium salt) (DPPG); the fluorescent dye 1,2-dipalmitoyl-sn-glycero-3-phosphoethanolamine-N-(lissamine rhodamine B sulfonyl) (ammonium salt) (DPPE-Rh) were purchased from Avanti Polar Lipids. Lipid solutions were prepared in chloroform and stored at −20 °C until use. DOPE-Atto647N was purchased from AtoTech. Glucose, sucrose, NaCl, CaCl_2_, EDTA, and the fluorescent probes Bodipy C_16_ (BODIPY FL C16; 4,4-Difluoro-5,7-Dimethyl-4-Bora-3a,4a-Diaza-s-Indacene-3-Hexadecanoic Acid), sulforhodamine B (SRB), Dextran-FITC 3 kDa, and bovine serum albumin (BSA) were purchased from Sigma-Aldrich. The small fluorescent KU530 NHS-Ester was purchases from KU dyes. Bodipy C_12_ was kindly provided by Klaus Suhling (King’s College London) and Gokhan Yahioglu (Antikor Biopharma).

GUVs were prepared using the polyvinyl alcohol (PVA) method (86) with minor modifications (60). In short, 100 μl of a 2% (weight/volume) PVA solution in water was spread on two glass coverslips and the water evaporated by placing the coverslip on a hot plate at ∼60 °C (typically 10 min) to form a PVA film. Next, a ∼10 μl lipid solution (2 mM) in chloroform containing the desired lipid mixture was spread on the PVA film and chloroform was evaporated under a stream of Argon. The two coverslips were sandwiched using a Teflon spacer forming a ∼2 ml chamber, which was then sealed with the help of clips. For GUV growth, the chamber was filled with a 200 mM sucrose solution (unless stated otherwise) for ∼30 min. If any of the lipids in the mixture had a Tm above room temperature (R.T = 18 ± 1 °C, if not stated otherwise), hydration was carried out in an oven at 50 to 60 °C, otherwise hydration was carried out at R.T. For lipid mixtures containing fluorescent lipids, hydration was performed in the dark. After hydration, the GUV solution was harvested by gentle pipetting and the GUVs were used within 1 day. For imaging, the GUVs were diluted in a solution containing isotonic 200 mM glucose to help sediment the vesicles to the bottom of the imaging chamber. Inner leaflet GUV mimics were made of DOPC:PS:Chol:DOPE (25:25:25:25, mol ratio – or DOPG instead of PS). Outer leaflet GUV mimics were made of SM:DOPC:PS:DOPE:Chol (24:30:0.6:5.4:40, mol ratio - or DOPG instead of PS). Alternatively, DOPG was used as an anionic lipid instead of PS. The GUVs were labeled with Bodipy C_16_ or Bodipy C_12_ at 0.5 mol% for the FRET or fluidity experiments, respectively.

The LUVs made of DOTAP:DOPE (1:1 mol ratio) labeled with 2 mol% DPPE-Rh were prepared using the hydration-sonication method (87). In short, the appropriate lipid mixture in chloroform was added to the bottom of a glass chamber and evaporated under a stream of Argon and further evaporated under vacuum for 1 to 2 h to remove any trace of chloroform. Next, the lipid film was hydrated with a 200 mM sucrose solution and vigorously vortexed until full lipid film detachment forming multilamellar vesicles. For the encapsulation of water-soluble probes, the reporter probe was prediluted in the hydrating sucrose solution. SRB was added at 50 μM, whereas Dextran-FITC was added at 0.1 mg/ml. If not stated otherwise, the final lipid concentration was 2 mM. The multilamellar vesicles were sonicated using a bath sonication for ∼20 min, which were ready for use and used within 2 to 3 days maximum. GUV incubation with the LUVs was done by diluting them in isotonic glucose. The LUVs were prediluted in sucrose to 100 μM lipid concentration and mixed with 50 μl GUVs in glucose at the desired final LUV concentration for a final 100 μl solution. GUVs and LUVs were incubated for 15 min. If the GUV lipids contained at least one lipid whose Tm was higher than R.T., incubation was done at 60 °C unless stated otherwise. For imaging, the incubated samples were moved to a BSA-coated glass (1 wt% BSA) coverslip. In the temperature-controlled experiments, a PeCon GmbH TempController 2000-1 temperature-controller was connected to a Heating Insert P Lab-Tek S heating stage chamber, into which the observation chamber was placed. For each temperature measurement, the sample was equilibrated for at least 5 min.

Membrane edge tension experiments were performed as in (56). In short, the GUVs were diluted ∼10× in isotonic glucose and placed in an electrofusion chamber containing two parallel cylinder electrodes (92 μm radius spaced by 500 μm (88)). The chamber was connected to an Eppendorf multiporator (Eppendorf), in which pulse strength and duration can be controlled from 50 to 300 V and 50 to 300 μs, respectively. A Zeiss Axiovert 200 (Jena) phase contrast microscope equipped with an sCMOS camera (pco.edge 4.2, PCO AG) for fast recordings (up to 300 frames per second, fps) was used. To record a typical event, higher magnifications (40 × or 63 × air objectives, NA 0.6 and 0.75, respectively) were employed. For membranes containing DO lipids, 150 V (3 kV/cm^−1^) field strength and 150 μs duration pulses were applied, whereas for the more viscous DP-containing membranes, 200 V (4 kV/cm^−1^) and 200 μs duration pulses were applied. Pore closure dynamics were analyzed by tracking pore sizes using PoET (57), a recently developed freely available Python-based software that measures edge tension from an automated fitting routine. Occasionally, the experiments were performed with small concentrations of salts (0.5 mM NaCl and 0.1 mM EDTA) in the outside medium to induce GUV oblate deformations (89) and remove CaCl_2_ contaminants.

Multicolor confocal microscopy imaging was performed on a Zeiss LSM 710 scanning confocal microscope. A C-Apochromat 40X/1.20 W Korr M27 water immersion objective was used for imaging. The spatial and temporal resolutions used were adjusted according to the sample conditions, but in general, a consistent imaging size of 212.55 μm × 212.55 μm (1024 × 1024 pixels) was used in a frame scanning mode in a singular direction, with a pixel size of 0.21 μm. Line averaging = 4 and bit depth = 8. The green dyes Bodipy C_12_, Bodipy C_16_, and Dextran-FITC 3 kDa were excited using a 488 nm argon laser, and emission was detected between 495 to 555 nm. The orange dye DPPE-Rh was excited with a 543 nm HeNe laser line and emission was detected in the range between 555 to 600 nm. The far-red dye Atto-647N was excited with a 633 nm HeNe laser line and its emission was detected in the range between 640 to 800 nm. Images were scanned in the sequential mode to minimize crosstalk between the different channels.

The FLIM experiments were carried out on an inverted microscope (Olympus IX73) equipped with time correlated single photon counting (PicoQuant). The samples were imaged through a 100× (1.4 NA) oil immersion objective (UPLSAPO, Olympus). Bodipy C_12_ was excited using a 481 nm laser and its emission was collected using a 525/50 nm band pass filter. The images were acquired using the SymPhoTime 64 software (PicoQuant) and all samples were excited with a pulsed 20 MHz repetition rate. Unless stated otherwise, the samples were imaged with a 128 × 128 pixels, 1 ms dwell-time, and ∼300 μm/pixels with typical acquisition times of ∼30 s. For analysis, the GUV membrane signal at the equator was manually selected for and all pixels binned for fitting. The fluorescence decays were fitted with a single (in the absence of an acceptor) or bi-exponential decay model (in the presence of the DPPE-Rh FRET acceptor)

I(t)=I0(A1e−tτ1+A2e−tτ2)

where *I*(*t*) is the intensity at time *t* and I_0_ is the intensity at *t* = 0. *A*_1_ and *A*_2_ are pre-exponentials factors associated with lifetime components *τ*_1_ and *τ*_2_, respectively (46).

The amplitude-weighted mean fluorescence lifetime in the presence of the FRET acceptor (*τ*) could be calculated as

$$\tau =A_{1}\tau_{1}+A_{2}\tau_{2}$$

To measure the FRET efficiency (E_FRET_), *τ* was obtained from GUVs both before (*τ*_*before*_) and after (*τ*_*after*_) incubation with the LUVs, in which FLIM-FRET efficiency is given as

EFRET=1−τafterτbefore

## Data availability

All data are contained in the manuscript and available upon reasonable request *via* direct contact with the corresponding author.

## Supporting information

This article contains supporting information.

## Conflict of interest

The authors declare that they have no conflicts of interest with the contents of this article.

## Supporting information

Movie S1 Electroporation of an Ld GUV made of DOPC:DOPG (5:5 mol ratio). GUV diameter before pulse: 38.5 μm. The onset of pulse application is detected at time = 28 ms. **Movie S2**Electroporation of an Ld GUV made of DOPC:DOPG:Chol (2:5:3 mol ratio). GUV diameter before pulse: 85.9 μm. The onset of pulse application is detected at time = 16 ms. **Movie S3**Electroporation of an Lo GUV made of DPPC:DPPG:Chol (2:5:3 mol ratio). GUV diameter before pulse: 39.8 μm. The onset of pulse application is detected at time = 36 ms. Note the wrinkles in the membrane due to high membrane viscosity. **Movie S4**Electroporation of an So GUV made of DPPC:DPPG (5:5 mol ratio). GUV diameter before pulse: 29.7 μm. The onset of pulse application is detected at time = 4 ms. Note that the formed pore remains indefinitely open during recording, through which intravesicular content is released.
